# Current Status of Herbal Medicines in Chronic Liver Disease Therapy: The Biological Effects, Molecular Targets and Future Prospects

**DOI:** 10.3390/ijms161226126

**Published:** 2015-12-02

**Authors:** Ming Hong, Sha Li, Hor Yue Tan, Ning Wang, Sai-Wah Tsao, Yibin Feng

**Affiliations:** 1 School of Chinese Medicine, Li Ka Shing Faculty of Medicine, The University of Hong Kong, Hong Kong, China; hong1986@connect.hku.hk (M.H.); lishasl0308@163.com (S.L.); hoeytan@connect.hku.hk (H.Y.T.); ckwang@hku.hk (N.W.); 2 Department of Anatomy, Li Ka Shing Faculty of Medicine, The University of Hong Kong, Hong Kong, China; gswtsao@hkucc.hku.hk

**Keywords:** chronic liver disease, herbal medicines, molecular targets, Chinese medicine herbal formulae

## Abstract

Chronic liver dysfunction or injury is a serious health problem worldwide. Chronic liver disease involves a wide range of liver pathologies that include fatty liver, hepatitis, fibrosis, cirrhosis, and hepatocellular carcinoma. The efficiency of current synthetic agents in treating chronic liver disease is not satisfactory and they have undesirable side effects. Thereby, numerous medicinal herbs and phytochemicals have been investigated as complementary and alternative treatments for chronic liver diseases. Since some herbal products have already been used for the management of liver diseases in some countries or regions, a systematic review on these herbal medicines for chronic liver disease is urgently needed. Herein, we conducted a review describing the potential role, pharmacological studies and molecular mechanisms of several commonly used medicinal herbs and phytochemicals for chronic liver diseases treatment. Their potential toxicity and side effects were also discussed. Several herbal formulae and their biological effects in chronic liver disease treatment as well as the underlying molecular mechanisms are also summarized in this paper. This review article is a comprehensive and systematic analysis of our current knowledge of the conventional medicinal herbs and phytochemicals in treating chronic liver diseases and on the potential pitfalls which need to be addressed in future study.

## 1. Introduction

Chronic liver diseases remain as one of the most serious health problems worldwide, which may affect more than 10% of the world population [1]. Chronic liver diseases in the clinical context describe pathological processes of the liver that involve a process of progressive destruction and regeneration of the liver parenchyma, which will finally lead to cirrhosis and hepatocellular carcinoma if left untreated. Among the various forms of chronic liver diseases, the most widely spread types include viral hepatitis, alcoholic or nonalcoholic fatty liver disease, autoimmune hepatitis, cirrhosis and hepatocellular carcinoma [2]. Excessive alcohol consumption, virus infection, obesity, diabetes and drug-induced liver damage are the leading causes of these liver diseases [3,4,5]. Although there have been remarkable progress in discovering treatment of chronic liver diseases over the last several decades, most of the therapies still do not yield satisfactory outcomes in patients [6]. In view of the scarce treatment options and significant adverse effects incurred by conventional chemical agents, novel prophylactic and therapeutic agents against chronic liver disease are urgently needed.

The use of herbal medicines can be traced back to more than 4000 years ago in ancient China. Over recent decades, an increasing number of herbal products, including medicinal herbs and phytochemicals, have been used for treating chronic liver diseases worldwide due to the high abundance, long-lasting curative effects and few adverse effects. According to the previous studies, medicinal herbs and phytochemicals could protect the liver by several mechanisms such as eliminating virus, blocking fibrogenesis, inhibiting oxidative injury and suppressing tumorigenesis [7,8]. As a chronic disease, most liver injuries need long-term treatment, thus, reducing side-effects of the therapy is critical when developing novel hepatoprotective agents. Although most of the patients believe that medicinal herbs and phytochemicals are natural and safe to be administrated without significant toxicity or side effects, all medicinal agents including herbal medicines potentially have toxicity and side effects. For safe use of medicinal herbs and phytochemicals, the potential side effects and toxicity of these hepatoprotective herbal medicines should be seriously taken into consideration. In this paper, we have reviewed several widely used and recognized medicinal herbs and phytochemicals in the present treatment of chronic liver diseases, including but not limited to *Coptis chinensis Franch* (berberine), *Glycyrrhiza uralensis Fisch* (glycyrrhizin), *Silybum marianum (L.) Gaertn.* (silymarine and silybinin), *Bupleurum chinensis DC* (saikosaponins), *Salvia miltiorrhiza Bunge* (salvianolic acid) and *Scutellaria baicalensis Georgi* (baiclin, wogonin). In addition, some Chinese medicine formulae for chronic liver disease treatment have also been reviewed in this paper. Based on the time-honored clinical experience in traditional Chinese medicine, it is believed that the multiple types of herbal or mineral ingredients in the formula could have multiple therapeutic molecular targets in chronic liver disease treatment. Both clinical trials and basic research of these herbal medicines were included to review the efficacy, potential molecular mechanisms as well as the side effects or toxicity of the active ingredients. In order to retrieve more recent publications about this topic, we conducted an updated search on the following databases from 1990 (one Chinese database and four English databases): China Journals Full-Text Database, MEDLINE, AMED (Allied and Complementary Medicine Database), EMBASE and The Cochrane Central Register of Controlled Trials (CENTRAL). Herbs and phytochemicals for chronic liver diseases treatment will be included in this review paper only if there are more than three papers describing the *in vitro* or animal study of the particular subject, or if any paper describing clinical trials on the subject. The inclusion criteria are stricter for traditional Chinese medicine formula as there are so many herbal formulae that have been applied in chronic liver disease treatment in traditional Chinese medicine. The included herbal formulae should have been studied by more than five original researches as well as at least one clinical trial which have demonstrated the potential therapeutic effects by this herbal formula in chronic liver diseases treatment.

## 2. Recent Research on the Roles of Herbs and Phytochemicals in Chronic Liver Disease Treatment

### 2.1. Herbs and Phytochemicals in the Treatment of Chronic Hepatitis

#### 2.1.1. The Epidemiological and Pathological Characteristics of Chronic Hepatitis and Current Therapeutic Strategy

Chronic hepatitis is the inflammation of the liver; the common causes of chronic hepatitis include viral infection, autoimmune diseases and toxic substances such as drugs or alcohol. Viral hepatitis is the most common liver diseases which may progress over time to fibrosis and cirrhosis [9]. The well-known pathogens for chronic viral hepatitis include Hepatitis viruses B (HBV) and Hepatitis viruses C (HCV). At present, more than 30% of the world’s population is infected with the HBV and 5% is considered as chronic HBV carriers. In some developed countries, due to the widespread vaccination of HBV, Hepatitis C has become the most common cause of viral hepatitis since the 1980s [10]. Hepatitis induced by toxins, such as alcohol or drugs, has also significantly increased in recent years. For alcoholic hepatitis, recent studies have indicated that younger people, females, and binge drinkers have a higher incidence of suffering from this disease and are associated with higher mortality rates [11]. A large number of drugs such as acetaminophen, antibiotics or other chemical agents are also common causes of hepatitis. Age, genetic variability, female sex, and previous drug-induced hepatitis are the key predisposing risk factors to drug-induced hepatitis [12]. The occurrence of autoimmune hepatitis is relatively rare; it is thought to have a genetic predisposition and is more likely to occur in young women. Patients with autoimmune hepatitis are often accompanied with other autoimmune diseases [13].

The specific mechanism of chronic hepatitis depends on the underlying causes of the disease. In viral hepatitis, the immune system is activated by the hepatic virus, resulting in inflammation and liver damage. In autoimmune hepatitis, the autoimmune disorder causes the abnormal immune response against liver cells. In alcoholic hepatitis, the damage is usually found in association with fatty liver which we will in a subsequent paragraph [14,15]. In general, the common characteristics of chronic hepatitis are inflammation, necrosis and fibrosis in liver tissues. The macrophages (Kupffer cells) and other liver-resident cells such as hepatic stellate cells and sinusoidal endothelial cells promote the formation and emitting of pro-inflammatory signals of cytokines, chemokines, lipid messengers and reactive oxygen species (ROS) and activate the inflammatory response that leads to hepatic cells oncotic necrosis and apoptosis [16,17,18]. Apoptotic bodies derived from the damaged hepatic cells can further activate Kupffer cells to secrete transforming growth factor beta 1, endothelial growth factor and platelet-derived growth factor, which can promote the transformation of activated hepatic stellate cells into myofibroblasts [19].

Currently, alpha-Interferon is the most widely used drugs for chronic Hepatitis B and C, although only a few patients can respond to interferon therapy. So far, several novel anti-viral agents such as nucleoside analogue have been applied to patients. However, these drugs cannot completely eradicate the hepatitis virus, while at the same time they may induce significant adverse effects and drug resistance [20]. For example, in patients with alcoholic hepatitis, pentoxifylline and prednisolone are the first-line recommended therapy. Unfortunately, a large proportion of patients do not respond to these drugs [21]. Budesonide with azathioprine may have potential therapeutic effects for autoimmune hepatitis, but the durability of response and target population still need further study [22]. Due to the limited therapeutic effect of conventional chronic hepatitis treatment, the use of complementary and alternative medicine is expanding throughout the world. In some Asian countries, people have treated chronic hepatitis with herbs or phytochemicals since ancient times; some herbal products have shown remarkable therapeutic effects in both basic and clinical studies (Table 1).

#### 2.1.2. Herbs and Phytochemicals in the Treatment of Chronic Hepatitis

Glycyrrhizin, an active component in *Glycyrrhiza uralensis Fisch*, has been used widely as a folk medicine agent for hepatitis in China and Japan. In Japan, glycyrrhizin injection has been used as an approved preparation for allergy inflammation since 1948 and for chronic hepatitis since 1979 [23]. Recently, glycyrrhizin has been found to suppress HCV particle release as well as the activity of phospholipase A2 (PLA2) [24]. As PLA2 has been proved to be related with HCV particles’ release, the researchers concluded that suppression of Hepatitis C by glycyrrhizin may be attributed to the reduced activity of PLA2. According to these studies, glycyrrhizin may be a promising agent for Hepatitis C when it is used alone or combined with interferon. According to the study by Fujisawa *et al.*, glycyrrhizin also showed significant anti-inflammatory effects in liver and other tissues. The mechanisms may be related to suppression of the cytolytic activity of complement by activating both classical and alternative pathways. Further studies demonstrated that glycyrrhizin suppresses the lytic pathway in which the membrane attack complex is formed [25]. *In vivo* studies also confirmed that glycyrrhizin can inhibit liver inflammation by enhancing the secretion of interleukin-10 (IL-10) in concanavalin-A (Con A)-induced hepatitis mice model which closely imitated the pathology of human autoimmune hepatitis. Further mechanism study revealed that glycyrrhizin can modulate the function of dendritic cells in mouse with autoimmune hepatitis and further promote the production of anti-inflammatory cytokines such as IL-10 [26]. There has a clinical case report on treating chemotherapy-induced HBV hepatitis with lamivudine combined with glycyrrhizin for a non-Hodgkin lymphoma patient. The result showed that glycyrrhizin combined with nucleotides’ analogue may be effective for controlling HBV replication in cancer patients [27]. Another clinical study in Europe also confirmed the therapeutic effect of glycyrrhizin in viral hepatitis. Sixty-nine out of 72 treatment courses were completed in this study. In the placebo group, there were no obvious changes of alanine aminotransferase (ALT) level. The mean percentage of ALT decrease by the end of treatment was 47% in the six-time glycyrrhizin treatment per week group and 26% for the three-time treatment per week group (*p* < 0.001). For the six-time per week treatment group, 20% (three of 15) patients got normal serum ALT levels and for the three-time per week treatment group, the result was about 10% (four of 41). No obvious side effects were detected in this study. The serum ALT levels went up again when treatment stopped [28].

Silymarin is a mixture of flavonolignans extracted from *Silybum marianum (L.) Gaertn* (milk thistle seeds) Major ingredients in silymarin include silibinin, isosilibinin, silicristin and silidianin. Silymarin is a time-honored herbal remedy for chronic liver diseases worldwide. Silibinin is the major active compound in silymarin, which has been widely studied for its hepatoprotective effects and anti-cancer effects both *in vitro* and *in vivo*. The main problems in the clinical application of silymarin include poor solubility and bioavailability. Recently, some modified derivatives of silymarin, such as the siliphos, have been developed for liver disease treatment. This new chemically synthesized agent contains lecithin which can significantly improve the solubility and bioavailability of silymarin [29]. Bonifaz *et al.* explored the antiviral effects of silymarin, and their results showed that silymarin can down-regulate the copy number of HCV core mRNA and protein expression. The antiviral mechanism of silymarin is different from interferon which inhibits the JAK/STAT signaling pathway [30]. Silymarin can block HCV entry and transmission by inhibiting the activity of microsomal triglyceride transfer protein and suppressing apolipoprotein B production. However, it seems that silymarin may not decrease the virus content *in vivo*, which has been proved by several clinical studies [31]. Mayer *et al.* developed a systematic review for the therapeutic effects of silymarin in treating chronic viral Hepatitis B and C, and the results demonstrated that silymarin can down-regulate serum transaminases in chronic viral hepatitis patients, but the liver histology or serum viral content have not been affected by silymarin [32]. A 12-month randomized controlled trial comparing the effects of silymarin with a multi-vitamin placebo in chronic HCV patients also showed that silymarin at the recommended dose has no better effect in clearing HCV RNA than the vitamin placebo. One hundred and fourty-one patients were evaluated in this study. After two years, 64 patients in the silymarin treatment group (*n* = 68) were HCV RNA positive and in 71 patients in the placebo group (*n* = 73) were HCV RNA positive (*p* = 0.61). Sixty-five patients in the silymarin treatment group were anti-HCV positive while 72 patients in the placebo group were anti-HCV positive (*p* = 0.56). [33]. Another clinical study explored the immunomodulatory properties of oral silymarin in patients with Hepatitis C, and their results revealed that silymarin can inhibit inflammation *in vitro* and *in vivo*. The mechanisms may be associated with inhibition of T-cell proliferation and production by suppressing the pro-inflammatory cytokine as well as up-regulating the anti-inflammatory IL-10 [34]. A meta-analysis demonstrated that silymarin combined with other anti-viral drugs may be beneficial for the chronic Hepatitis B patients [35]. Silibinin, the major active ingredient in silymarin, also exhibited the inhibitory effects on Hepatitis C virus by blocking the clathrin-dependent trafficking. Silibinin can influence the formation of both clathrin-coated pits and vesicles in liver cells and destroy the clathrin endocytic pathway by interfering with the absorption and trafficking of transferrin [36]. A short-term pilot study on 20 patients with chronically active hepatitis was carried out to assess the liver protective activity and the antioxidant properties of a new silybinin phosphatidylcholine complex, and the results showed that after treatment with this new silybinin complex, there was a remarkable reduction of serum aspartate transaminase (AST) from 88.0 (95% CI 65.52–113.48) to 65.9 (95% CI 52.23–78.57) µ/L, (*p* < 0.01), a decrease of ALT from 115.9 (95% CI 94.1–147.7) to 82.5 (95% CI 64.59–100.41) µ/L (*p* < 0.01), and a decrease of total bilirubin from 0.76 (95% CI 0.63–0.89) to 0.53 (95% CI 0.47–0.59) mg/dL (*p* < 0.05) and of gamma-glutamyltranspeptidase from 51.4 (95% CI 35.69–67.11) to 41.3 (95% CI 36.01–50.19) µ/L (*p* < 0.02). Decrease of Alkaline phosphatase was not obviously, from 143.4 (95% CI 132.59–154.21) to 137.5 (95% CI 124.32–150.68) µ/L [37].

*Phyllanthus niruri L* is a subtropical plant widely distributing in China and South Asia, which has been used as traditional medicines to treat chronic hepatitis as well as intestinal infections and kidney disease [38,39,40]. The main active compounds in *Phyllanthus niruri L* including quercetin rhamnoside, gallic acid, geraniin and quercetin glucoside have been identified and proved with the properties of anti-bacterial, anti-viral and anti-hepatotoxic effects [41]. In recent years, several researches for evaluating the anti-viral effects and safety of *Phyllanthus niruri L* have been conducted both *in vitro* and *in vivo*. Lam *et al.*, found that the ethanolic extract of *Phyllanthus niruri L* could suppress HBsAg secretion and down-regulate the expression of HBsAg mRNA, with a mechanism that may be related to up-regulation of annexin A7 protein [42]. A systematic review of 22 randomized trials (*n* = 1,947) on using *Phyllanthus niruri L* for treating chronic Hepatitis B showed that *Phyllanthus niruri L* were effective in clearance of serum HBsAg, HBeAg and HBV DNA without serious side-effects. When compared with interferon (IFN) and placebo, the therapeutic effects of combination treatment of *Phyllanthus niruri L* and IFN was better [43].

The rhizome of *Polygonum cuspidatum Willd. ex Spreng.* has been used as folk medicine for treating chronic hepatitis, jaundice, cough, hyperlipidemia and arthralgia in some Eastern Asian countries for thousands of years. This herbaceous perennial plant is widely distributed in the world and prefers to grow in humid environment such as the valley or forest [44]. Recent pharmacological studies have shown that *Polygonum cuspidatum Willd. ex Spreng.* has antiviral and hepatoprotective effects, and the major active ingredients may include anthraquinones, resveratrol and polydatin [45]. According to a recent meta-analysis review study, *Polygonum cuspidatum Willd. ex Spreng.* is one of the most frequently used herbs in the traditional Chinese medicine for chronic hepatitis treatment [46]. *In vitro* studies also confirmed that the water extract of *Polygonum cuspidatum Willd. ex Spreng.* at the concentration of 30 μg/mL could suppress the expression of Hepatitis B e antigen (HBeAg). For the ethanol extract of *Polygonum cuspidatum Willd. ex Spreng.*, it could inhibit the production of HBV DNA at a low dose (10 μg/mL) [47].

*Bupleurum chinense DC* is one of the most frequently used herbs for relieving exterior syndrome in traditional Chinese medicine. When applying bupleurum decoction in combination with long-term exercise training, rats with obstructive jaundice exhibited a significant reduction in inflammatory cytokines expression, which in turn alleviated the liver hepatitis [48]. The leaf of Bupleurus has been regarded as the useless part in Chinese medicine, but a recent study has demonstrated that the saikosaponins extracted from Bupleurus leaves exhibit significant antioxidant effects and hepatoprotective activity. *In vitro* study proved that the saikosaponins exhibited free radical scavenging activity in diphenyl picryl hydrazinyl radical and can suppress the superoxide anion formation and the activity of superoxide anion scavenging in liver cells [49]. There are many types of saikosaponin and each type may have different biological effects *in vivo*. Previous study on saikosaponin C has found that the HBV-infected liver cells cultured with saikosaponin C exhibited a remarkable lower expression of HBV antigen in culture medium, and this antiviral effect was not due to the cytotoxicity of saikosaponin C [50]. Saikosaponin B2 was also an effective antiviral agent. *In vitro* study confirmed that saikosaponin B2 can suppress early HCV entry, such as neutralization of virus particles, inhibiting viral attachment, preventing viral fusion, and finally blocking HCV infection of primary human hepatocytes [51].

*Salvia miltiorrhiza Bunge*, also known as Chinese sage, has been widely used for the treatment of cardiovascular and cerebrovascular diseases. Recent studies found the *Salvia miltiorrhiza Bunge* can increase blood flow into the liver to reduce the potential damage by clearing the harmful substance in the liver. Active ingredients of *Salvia miltiorrhiza Bunge* can also exert hepatoprotective effect from CCl_4_-induced liver injury in rats. The protective effect may be due to inhibition of NF-kB and p38 signaling in liver Kupffer cells [52]. Treatment of chronic iron-overloaded mice with *Salvia miltiorrhiza Bunge* will improve the hepatic morphology, decreasing iron deposition as well as inhibiting the expression of type I and type III collagen, TGF-beta mRNA, and increase the expression of matrix metalloprotein-9 (MMP-9) mRNA in the liver. Moreover, *Salvia miltiorrhiza Bunge* treatment can decrease malondialdehyde content as well as increase glutathione (GSH) content and superoxide dismutase (SOD) activity while it reduced expression of TNF-alpha, IL-1alpha and caspase-3 [53].

Matrine and oxymatrine are both alkaloids with antiviral and anti-inflammation effects, which can be isolated from the herb of *Sophora flavescens Aiton*. Ma *et al.* found that the single use of oxymatrine (100 mug/mL), matrine (200 mug/mL), and the nucleoside analogs lamivudine (30 mug/mL) did not show any inhibition effects on the content of HBsAg, HBeAg, and HBV-DNA in culture media. However, the combination of lamivudine (30 mug/mL) with oxymatrine (100 mug/mL) or matrine (100 mug/mL) can significantly suppress the content of HBsAg, HBeAg, and HBV-DNA, the effects of combination treatment were even more remarkable than that from using lamivudine alone at 100 mug/mL [54]. A recent study has analyzed the molecular mechanisms underlying matrine’s anti-inflammatory effects in hepatic cells. The results found that matrine could increase the concentration of nitric monoxide (NO) in culture supernatant of rat intestinal microvascular endothelial cells (RIMECs) in a dose-dependent manner and up-regulate the endothelial nitric oxide synthase (eNOS) concentration, which further improves the vasomotion in liver tissue. Additionally, matrine reduced the secretion of IL-8, IL-6, and sICAM-1 induced by lipopolysaccharide (LPS) in RIMECs. These results indicated that matrine could inhibit inflammation in liver and might be beneficial for patients with hepatitis [55]. Yao *et al.* investigated the anti-HBV immunomodulatory mechanism of oxymatrine *in vitro*. Their results showed that oxymatrine could activate the peripheral lymphocytes and induce antiviral cytokine secretion. Pretreatment with oxymatrine could modulate toll-like receptor 9(TLR9) signal pathway and promote the immune function of the TLR9 ligand against virus [56].

Periplocoside A (PSA), a pregnane glycoside, is a new phytochemicals isolated from *P. sepium Bge* which is widely used for treating rheumatoid arthritis in traditional Chinese medicine. A recent study has examined the protective effects of PSA on T cell-mediated hepatitis induced by concanavalin A (ConA) in murine model. Pretreatment with PSA significantly ameliorated liver damage induced by ConA, the secretion of interleukin (IL)-4, interferon (IFN)-gamma and serum alanine transaminase (ALT) levels were dramatically decreased, which further inhibited the hepatocyte necrosis and protected the liver function *in vivo*. *In vitro* studies demonstrated that PSA suppressed the secretion of inflammatory cytokines such as IL-4 and (IFN)-gamma produced by Natural killer T cells upon stimulation with anti-CD3 mAb or α-galactosylceramide. In addition, no obvious toxicity has been observed both *in vitro* and *in vivo* with PSA treatment. These results suggested that the new phytochemicals PSA may have therapeutic potential for treating human autoimmune-related hepatitis [57,58].

Baicalin, which is extracted from *Scutellaria baicalensis Georgi*, was proved to be able to protect hepatocytes from oxidative stress by up-regulating both liver fatty acid binding protein expression and activity of intracellular SOD and GSH [59]. Animal experiments confirmed that baicalin presents anti-inflammatory, anti-oxidant, and anti-apoptotic effects, which offered its hepatoprotective effects against hepatocellular ischemia/reperfusion-induced injury [60]. Wan *et al.* found that 50-day treatment of the baicalin-containing diet (0.25% and 1%) also exhibited protective effects on liver injury in iron overload mouse, the mechanism of which may be associated with the iron chelation and antioxidant activities of baicalin [61]. Baicalein, another active compound isolated from *Scutellaria baicalensis Georgi*, may be a promising agent for liver injury. According to a recent study, it can accelerate liver cells’ regeneration by modulating IL-6 and TNF-alpha mediated signaling pathways [62]. Another study found that the molecular mechanisms involved in baicalein-inhibited apoptosis of liver cells were the protective effect on mitochondria, inhibition of the release of cytochrome c, decrease of the Bax/Bcl-2 ratio, and suppression of the phosphorylation of NF-kB, JNK and ERK [63].

*Schisandra chinensis (Turcz.) Baill* is a frequently used herbal medicine in China, where it has a common name *Wuweizi* (five-flavor berry). According to the theory of traditional Chinese medicine, *Schisandra chinensis (Turcz.) Baill* can supplement Qi and promote the production of body fluid. Modern pharmacological studies confirmed that *Schisandra chinensis (Turcz.) Baill* may regulate the body's humoral balance, promote cell-mediated immune responses and has anti-HBV properties [64]. Xue *et al.* isolated and investigated seven unknown lignans and a schischinone, together with several known lignans from the fruits of *Schisandra chinensis (Turcz.) Baill*. Their study found that two lignans from the *Schisandra chinensis (Turcz.) Baill* exhibited significant anti-Hepatitis B virus activity by suppressing HBV DNA replication without obvious cell toxicity on hepatic cells [65]. Schisandrin B, a dibenzocyclooctadiene derivative isolated from the fruit of *Schisandra chinensis (Turcz.) Baill*, has shown therapeutic efficiency for the treatment of hepatitis. The anti-inflammatory mechanisms of Schisandrin B were investigated by Checker *et al.* The results showed that Schisandrin B can induce nuclear translocation of redox sensitive nuclear factor erythroid-derived factor 2-related factor (Nrf2) and increase the transcription of Heme Oxygenase-1(HO-1). Schisandrin B can also inhibit the nuclear translocation of NF-kB in activated lymphocytes and suppress the expression of downstream inflammation-related genes [66].

*Astragalus membranaceus (Fisch.) Bunge* has been one of the most widely used herbs in China for thousands of years. According to the traditional Chinese medicine theory, it could increase metabolism, improve the functions of lungs and the gastrointestinal tract, promote wound healing and provide fatigue relief. The main active compounds in *Astragalus membranaceus (Fisch.) Bunge* are flavonoids and saponins, such as calycosin, formononetin, astragaloside, and calycosin-7-*O*beta-D-glucoside [67,68]. Modern researches have shown that *Astragalus membranaceus (Fisch.) Bunge* could modulate immunologic system, inhibit virus growth and suppress cancer proliferation [69,70]. A recent study has indicated that *Astragalus membranaceus (Fisch.) Bunge* has potential therapeutic effects in patients with viral hepatitis. *In vivo* studies showed that *Astragalus membranaceus (Fisch.) Bunge* could suppress duck HBV DNA replication. The liver pathological changes in duck viral Hepatitis B models with *Astragalus membranaceus (Fisch.) Bunge* treatment were also milder than the control group. Clinical study was conducted by the same group, in which *Astragalus membranaceus (Fisch.) Bunge* was used for treating 116 patients with chronic viral hepatitis while 92 patients were given regular antiviral agents. The proportion of negative seroconversions of HBeAg and HBV DNA were significantly higher in the *Astragalus membranaceus (Fisch.) Bunge* group than in the control group (*p* < 0.01 and *p* < 0.05 respectively). These results showed that the clearance of serum HBeAg and HBV DNA was better in *Astragalus membranaceus (Fisch.) Bunge* treatment group [71]. Further studies have identified that the active ingredients in *Astragalus membranaceus (Fisch.) Bunge* for inhibiting HBV activities may be triterpenoid saponin [72].

**Table 1:** Herbs and phytochemicals in chronic hepatitis treatment.

Herbal Medicines (Herbs or Phytochemicals)	The Sources of Herbal Medicines	Type of Study	Biological Effects and Molecular Mechanism	Reference
Glycyrrhizin	Root of *Glycyrrhiza uralensis Fisch*	*In vitro*	Suppression of Hepatitis C by decreasing the activity of phospholipase A2.	[24]
		*In vivo*	Suppressing the cytolytic activity of complement.	[25]
		Clinical study	Enhancing the secretion of IL-10 by dendritic cells.	[26]
			Controlling HBV replication.	[27]
			Decreasing serum ALT.	[28]
Silibinin	Fruits *of Silybum marianum (L.) Gaertn*	*In vitro*	Inhibiting HCV by blocking the clathrin-dependent trafficking.	[36]
		Clinical study	Decreasing the secretions of ALT, AST and alkaline phosphatase in patients with chronic active hepatitis.	[37]
Saikosaponin C, Saikosaponin b2	Root of *Bupleurum marginatum Wall. ex DC*	*In vitro*	Decreasing HBV antigen in culture medium.	[50]
			Suppressing early HCV entry.	[51]
Matrine, Oxymatrine	Root of *Sophora flavescens Aiton*	*In vitro*	Improving the effect of lamivudine on suppressing the secretion of HBeAg in combination with oxymatrine or matrine,	[54]
		*In vivo*	Improving the vasomotion in liver tissue by increasing the concentration of NO in culture supernatant of rat intestinal microvascular endothelial cells and up-regulating the eNOS concentration.	[55]
		*In vivo*	Activating the peripheral lymphocytes and inducing antiviral cytokine secretion by regulating TLR9 signal pathway.	[56]
Periplocoside A	Bark of *Periploca sepium Bunge*	*In vitro* and *in vivo*	Ameliorating autoimmune hepatitis induced by ConeA by decreasing the secretions of (IL)-4, IFN-gamma and ALT.	[57]
Baicalin, Baicalein	Root of *Scutellaria baicalensis Georgi*	*In vitro*	Protecting hepatocytes from oxidative stress by up-regulating both liver fatty acid binding protein expression and activity of intracellular SOD and GSH.	[59]
			Anti-inflammatory, anti-oxidant, and anti-apoptotic in hepatic cells.	[60]
		*In vivo*	Iron chelation and antioxidant effects in iron overload liver.	[61]
			Accelerating liver cells regeneration by modulating IL-6 and TNF-alpha mediated signal pathways.	[62]
		*In vitro*	Inhibiting apoptosis in liver cells by the protective effect on mitochondria, inhibiting the release of cytochrome c, decreasing the Bax/Bcl-2 ratio, and inhibiting the phosphorylation of NF-kappaB, JNK and ERK.	[63]
Schisandrin B	Fruit of *Schisandra chinensis (Turcz.) Baill*	*In vitro*	Anti-inflammation in liver by inducing nuclear translocation of Nrf2 and increasing the transcription of HO-1.	[66]
Silymarin	Flavonolignans mixture from the fruits of *Silybum marianum (L.) Gaertn.*	*In vitro*	Down-regulating the HCV core mRNA and protein expression.	[30]
			Blocking of HCV entry and transmission by inhibiting microsomal triglyceride transfer protein activity, apolipoprotein B secretion, and infectious virion production into culture supernatants.	[31]
		Clinical study	Decreasing serum transaminases in patients with chronic viral hepatitis, but not affecting viral content *in vivo.*	[32,33]
			Inhibiting inflammatory by suppressing the pro-inflammatory cytokine and up-regulating the IL-10.	[34]
*Salvia miltiorrhiza Bunge*	Aqueous extract of root and rhizome of *Salvia miltiorrhiza Bunge*	*In vivo*	Reducing inflammation in the liver by inhibition of NFkappaB and p38 signaling.	[52]
			Improving the hepatic morphology, decreasing iron deposition as well as inhibiting the expression of type I and type III collagen, TGF-beta mRNA, and increasing the expression of MMP-9 mRNA in the liver.	[53]
*Bupleurum chinense DC*	Aqueous extract of root or leaf of *Bupleurum chinense DC*	*In vitro*	Scavenging free radical activity and suppressing the superoxide anion formation.	[49]
		*In vivo*	Reducing inflammatory cytokine expression.	[48]
*Polygonum cuspidatum Willd. ex Spreng.*	Ethanol extract of rhizome of *Polygonum cuspidatum Willd. ex Spreng.*	*In vitro*	Suppressing the expression of HBeAg and the production of HBV DNA.	[47]
*Schisandra chinensis (Turcz.) Baill*	Aqueous extract of fruit of *Schisandra chinensis (Turcz.) Baill*	*In vitro*	Anti-Hepatitis B virus activity by suppressing HBV DNA replication.	[65]
*Phyllanthus niruri L*	Aqueous extract of whole plant of *Phyllanthus niruri L*	*In vitro*	Inhibiting HBsAg secretion and HBsAg mRNA expression by up-regulation of annexin A7.	[42]
		Clinical study	Clearance of serum HBsAg, HBeAg and HBV DNA.	[43]
*Astragalus membranaceus (Fisch.) Bunge*	Aqueous extract of root of *Astragalus membranaceus (Fisch.) Bunge*	*In vivo*	Suppressing duck HBV DNA replication.	[72]
		Clinical study	Clearance of serum HBeAg and HBV DNA in chronic viral hepatitis patients.	

### 2.2. Herbs and Phytochemicals in the Treatment of Fatty Liver Disease

#### 2.2.1. The Epidemiological and Pathological Characteristics of Fatty Liver Disease and Current Therapeutic Strategy

Fatty liver disease, or steatosis, is a reversible pathological process wherein large vacuoles of triglyceride fat accumulate in liver cells. Fatty liver disease is usually induced by excessive alcohol consumption or metabolic disorders such as obesity, insulin resistance and dyslipidemia [73]. Alcoholic fatty liver (AFL) refers to the alcohol-related steatosis while nonalcoholic fatty liver (NAFL) means the original cause of steatosis is not alcohol. In Western countries, fatty liver disease is the most common liver disease, the prevalence of which ranges from about 20%–60% [74]. In 1992, some researchers in Italy started the Dionysos Study that intended to evaluate the prevalence and incidence of chronic liver disease in two communities of Northern Italy. According to their results, 45% of the residents had fatty liver disease identified by liver ultrasonography. As fatty liver diseases cannot be distinguished at liver biopsy, normally, their distinction depends on alcohol consumption. Using a cut-point of 20 g/d for ethanol consumption, about 25% residents had NAFL while 20% had AFL [75]. In patients with NAFL, approximately 30% patients will progress to non-alcoholic steatohepatitis (NASH). In those with NASH, approximately 20% will further progress to cirrhosis, in which the histological lesions of the liver are irreversible. For patients with AFL, nearly 20%–40% of these patients will progress to alcoholic steatohepatitis (ASH). Of those with ASH, approximately 40% will develop cirrhosis [76].

Defects in fatty acid metabolism are responsible for the initial stage of fatty liver disease, which may lead to lipid overstorage in the liver. For AFL, alcoholism may damage mitochondria and other cellular structures, and further leads to cellular fatty acid metabolism disorder. For NAFL, insulin resistance and other metabolism disorder induced the lipid storage and progress to steatosis. In both AFL and NAFL, steatosis will sensitize the liver tissue to the induction of inflammation by oxidative stress and will further develop steatohepatitis [77]. Actually, only a part of patients with steatosis will progress to steatohepatitis, and the mechanisms are still unclear. The inflammation and a high degree of steatosis will induce hepatocyte necrosis, lipid peroxidation and activation of stellate cells, which play an important role in liver fibrosis [78].

The therapeutic strategy for fatty liver depends on its cause, treating the underlying cause at an early stage of steatosis may reverse the process and inhibit steatohepatitis. Thus, strict restriction of alcohol consumption and avoiding high calorie containing foods are important interventions for patients with fatty liver. For those with early stages of non-alcoholic fatty liver disease, a gradual weight loss is the only solution for preventing disease progression. Conventional medications such as ursodeoxycholic acid, insulin sensitizers, antioxidants or lipid-lowering drugs for decreasing insulin resistance, hyperlipidemia, and inducing weight loss may have certain therapeutic effects on pure steatosis without inflammation. For advanced patients with steatohepatitis, there are still no effective treatments [79,80]. According to recent studies, some herbal products seemed to have positive effects on this potentially reversible disease. Both basic and clinical studies suggested that herbal medicines may have modest benefit for fatty liver disease treatment (Table 2).

#### 2.2.2. Herbs and Phytochemicals in the Treatment of Fatty Liver Disease

Berberine is a quaternary ammonium salt isolated from the plant of Berberis such as *Coptis chinensis Franch*, which is one of the 50 fundamental herbs used in traditional Chinese medicine. The alkaloids in *Coptidis Rhizoma* aqueous extract (CRAE) were found to be effective in reducing triglyceride (TG) accumulation in the FFA-induced hepatic steatosis in liver cells. Berberine has been confirmed as the major compartment in inhibition of TG accumulation [81]. Berberine can also enhance insulin resistance of nonalcoholic fatty liver disease by increasing the expression of insulin receptor substrate-2 (IRS-2) and regulating the insulin signaling pathway [82]. Xue *et al.* found that the berberine-loaded solid lipid nanoparticles (BBR-SLNs) can relieve hepatosteatosis in db/db mice by suppressing lipogenesis and promoting lipolysis in the liver. Further study of the mechanisms revealed that BBR-SLNs can inhibit the expression of lipogenic genes such as stearoyl-CoA desaturase (SCD1), fatty acid synthase (FAS), and sterol regulatory element-binding protein 1c (SREBP1c), and increase the expression of lipolytic gene such as carnitine palmitoyltransferase-1 (CPT1) [83].

*Gynostemma pentaphyllum (Thunb.) Makino* (GP) is a dioecious, herbaceous climbing vine distributed in Eastern Asian. It is widely used for lowering cholesterol, and preventing fatty liver and obesity in China and Japan. Wang *et al.* found that GP extracts can promote lipid metabolism, and decrease serum lipids level by down-regulating the production of trimethylamine N-oxide (TMAO) and up-regulating the secretion of phosphatidylcholine [84]. In a study with a NAFL cellular model, GP extract was efficient in inhibiting the accumulation of cholesterol and triglycerides as well as preventing oxidative stress in liver cells. Further study of the mechanisms revealed that GP enhances the production of nitric oxide (NO) which reduces oxidative stress damage in liver cells and affects the molecular composition of the mitochondrial phospholipid cardiolipin (CL) [85]. A randomized, single-blind, controlled clinical trial showed that six-month dietary GP treatment can significantly reduce the level of serum AST, ALP, insulin, and decrease the body mass index (BMI) and insulin resistance index. In the GP group, BMI had reduced remarkably from 28.4 at month 0 to 27.5 at month 6 (*p* = 0.018), total cholesterol had dropped from 228.5 (95% CI 148.06–308.94) to 206.1 (95% CI 139.35–272.85) (*p* = 0.004), and ALT had decreased from 80.2 (95% CI 19.53–140.87) to 63.2 (95% CI 3.99–122.51) (*p* = 0.004) [86]. These studies demonstrated that GP extracts can be an effective adjunct treatment for NAFL patients. Ombuine is a flavonoid isolated from GP. Recent studies confirmed that ombuine treatment remarkably decreased intracellular concentrations of triglyceride and cholesterol in liver cancer cells and reduced the expression of some lipogenic genes through activating PPARα and β/δ. Furthermore, Ombuine can also decrease cellular cholesterol concentrations by activating ATP binding cassette cholesterol transporter A1 and G1 protein expression [87].

*Panax notoginseng (Burkill) F.H.Chen* (PNS) is a species of the genus *Panax*, which grows naturally in China and Japan. Ding *et al.* found that PNS extracts can attenuate the ethanol induced hepatic lipid accumulation by inhibiting the production of malondialdehyde (MDA), glutathione (GSH) l and reactive oxygen species (ROS), reducing TNF-alpha and IL-6 levels, as well as enhancing the superoxide dismutase (SOD) activity in liver, and abrogating cytochrome P450 2E1 (CYP2E1) induction. In addition, PNS can protect liver cells from CYP2E1-mediated oxidative stress [88,89]. *In vivo* studies from the same group also found that total saponins of PNS can relieve oxidative stress and insulin resistance in NAFLD rat models. Concentrations of malondialdehyde (MDA), the hydroxy radical level (–OH), and TNF-alpha decreased after PNS treatment, while the activities of total superoxide dismutase (T-SOD) and total antioxidant capacity (T-AOC) were recovered, and insulin resistance improved significantly.

Penta-oligogalacturonide is isolated from the fruit of *Crataegus pinnatifida Bunge*, which is the most often used herb in the treatment of NAFLD according to the systematic review study by Shi *et al.* [90]. Recently, the anti-lipidemic and antioxidant effects of penta-oligogalacturonide were investigated both *in vitro* and *in vivo*. The results showed that penta-oligogalacturonide was effective in scavenging hydroxyl, superoxide anion and 2,2-diphenyl-1-picrylhydrazyl (DPPH) radicals in liver cells. Additionally, penta-oligogalacturonide can enhance the antioxidant enzyme activities of superoxide dismutase, catalase, and glutathione peroxidase, increase the levels of glutathione and the total antioxidant capacity, but lower the production of malondialdehyde in the liver of high-fat fed mice. Furthermore, penta-oligogalacturonide significantly decreased the triglyceride levels, expression of phosphatidate phosphohydrolase (PAP) and glycerol 3-phosphate acyltransferase (GPAT) in mice livers. Moreover, the liver biopsy results confirmed that penta-oligogalacturonide treatment at a dose of 300 mg/kg can inhibit liver steatosis in mice with high-fat diet [91]. Another study from the same research group also found that penta-oligogalacturonide remarkably increased the liver fatty acid oxidation-related enzyme activities of acyl-CoA oxidase, 3-ketoacyl-CoA thiolase, 2,4-dienoyl-CoA reductase, and carnitine palmitoyltransferase I, which may prevent hyperlipemia in the liver of high fat diet mice [92].

Dioscin, a natural steroid saponin isolated from *Dioscorea opposita Thunb*, has been shown to have a remarkable protective effect against fatty liver disease. Recent study has demonstrated that dioscin can reduce body weight and lipid accumulation in liver, increase oxygen consumption and energy expenditure and decrease the levels of serum ALT, AST. Further study of the mechanisms revealed that dioscin can relieve oxidative damage, inhibit inflammation, suppress cholesterol and triglyceride synthesis, decrease mitogen-activated protein kinase (MAPK) phosphorylation levels, promote fatty acid beta-oxidation, and induce autophagy to improve fatty liver conditions. These results indicated that dioscin may be a potential candidate for obesity and NAFLD prevention [93]. Dioscin also showed an excellent protective effect against alcoholic fatty liver by relieving alcohol-induced oxidative stress, inflammatory cytokine production, mitochondrial function, apoptosis and liver steatosis *in vivo* [94].

Gallic acid is a trihydroxybenzoic acid found in the pericarp of *Punica granatum L*. Previous studies have confirmed that gallic acid has potent anti-oxidative and anti-obesity activity. Chao *et al.* found that gallic acid can improve the glucose and lipid metastasis disorder in NAFLD mice. The hepatoprotective effect by gallic acid may be related to regulation of the metabolism of choline, amino acids, glucose, lipid and gut-microbiota in mice [95]. Histological study in the high fat diet-induced NAFLD rat showed that the lipid droplets in gallic acid treatment group were significantly smaller than the control group. When compared with the control group, cholesterol level and hepatic triacylglycerol (TAG) in gallic acid treatment groups were remarkably reduced. Furthermore, gallic acid treatment decreased oxidized glutathione (GSSG) content and increased the levels of anti-oxidative enzyme in liver. These results indicated that gallic acid treatment can inhibit NAFLD-related hepatosteatosis, dyslipidaemia and oxidative stress in rats [96].

Compound glycyrrhizin has been widely used in clinical practice for chronic liver disease. An *in vivo* study has proved the therapeutic effects of compound glycyrrhizin liposome on nonalcoholic steatohepatitis in rats in inhibiting the process of fibrosis [97]. Another study also confirmed that the glycyrrhizin-containing preparation can suppress the development of hepatic steatosis in a dose-dependent manner and attenuate ultrastructural alterations of mitochondria of the hepatocytes. In addition, it can down-regulate the reactive oxygen species in mice liver [98]. These results demonstrated that the glycyrrhizin-containing preparation can prevent hepatic steatosis possibly by preventing mitochondria damage from oxidative stress.

According to previous studies, the curative effects of silymarin in alcoholic and non-alcoholic steatohepatitis are widely recognized [99]. Recently, siliphos, a modified derivative of silymarin, has been proved to effectively suppress severe oxidative stress and to protect hepatic mitochondrial bioenergetics in nonalcoholic steatohepatitis [100]. Through protecting mitochondrial function, the damage of lipid membranes by oxidation is alleviated by siliphos. Clinical trials also confirmed that after siliphos treatment, ultrasonographic score for liver steatosis has decreased remarkably in patients with nonalcoholic fatty liver disease. In addition, a significant improvement in liver enzyme levels, indexes of liver fibrosis, and hyperinsulinemia have been observed in siliphos treatment group [101].

The kernels of *Prunus armeniaca L* are used as a common drug in traditional Chinese medicine. As a popular fruit, dietary intake of *Prunus armeniaca L* kernels can lower cholesterol levels in the body [102]. Recent studies have found that feeding rats with the kernels of *Prunus armeniaca L* had protective effects against CCl_4_-induced liver steatosis due to its remarkable antioxidant and radical-scavenging capacity. The rich content of beta-carotene and vitamin may be associated with the anti-steatosis effect by the kernels [103]. In a clinical study, the extract of *Prunus armeniaca L* kernels also exhibit protective effects on fatty liver disease patients. The results showed that serum ALT and AST levels were remarkably reduced compared with pre-intake baseline levels from 103.5 (95% CI 4.03–202.87) IU/L to 71.8 (95% CI 5.39–138.21) IU/L (*p* < 0.05) and from 93.5 (95% CI 0.02–187.72) IU/L to 65.5 (95% CI 6.69–123.31) IU/L (*p* < 0.05). A decrease of more than 30% from the pre-study baseline ALT and AST levels were detected in 45% and 43% of the patients (*n* = 58), respectively. These results indicated that after treating these patients with *Prunus armeniaca L* extracts, the serum AST and ALT levels were significantly decreased compared with the control group [104].

Baicalin was also found to be effective in modulating lipid metabolism and suppressing systemic inflammation in high-fat feeding rat liver and the mechanism may be related to the regulation of AMPK-alpha signaling pathway [105,106]. Further study confirmed that baicalin can decrease the levels of serum total cholesterol, triglycerides, low density lipoprotein (LDL), ALT and AST, and increase the levels of serum HDL. Pathological analysis revealed a higher dose of baicalin diminished both macrovesicular and microvesicular steatoses while a low dose of baicalin only suppressed macrovesicular steatosis. The mechanism may be associated with the regulation of hepatic CaMKKbeta/AMPK/ACC pathway [107]. For alcoholic fatty liver disease, baicalin is also effective in attenuating the ischemia/reperfusion injury in alcoholic fatty liver. In ethanol-fed animals, baicalin attenuated inflammatory responses by suppressing of myeloid differentiation factor 88 and toll-like receptor 4 expressions and the nuclear translocation of NF-kB after reperfusion [108].

**Table 2:** Herbs and phytochemicals in fatty liver disease treatment.

Herbal Medicines (Herbs or Phytochemicals)	The Sources of Herbal Medicines	Type of Study	Biological Effects and Molecular Mechanism	Reference
Berberine	Rhizoma of *Coptis chinensis Franch*	*In vitro*	Reducing TG accumulation in the FFA-induced hepatic steatosis.	[81]
		*In vivo*	Enhancing insulin resistance of nonalcoholic fatty liver disease by increasing the expression of IRS-2.	[82]
			Suppressing lipogenesis and promoting lipolysis by inhibiting the expression of SCD1, FAS, SREBP1c and increasing the expression of CPT1.	[83]
Ombuine	Whole plant of *Gynostemma pentaphyllum (Thunb.) Makino*	*In vitro*	Reducing intracellular concentrations of triglyceride and cholesterol in HepG2 cells and decreased the expression of several lipogenic genes by activating of PPARalpha and delta/beta.	[87]
Penta-oligogalacturonide	Fruit of *Crataegus pinnatifida Bunge*	*In vitro*	Scavenging hydroxyl, superoxide anion and DPPH radicals in liver cells.	[92]
		*In vivo*	Enhance the antioxidant enzyme activities of superoxide dismutase, catalase, glutathione peroxidase, increase the levels of glutathione and the total antioxidant capacity, but lowered the production of malondialdehyde in the liver of high-fat fed mice.	
Glycyrrhizin	Root of *Glycyrrhiza uralensis Fisch*	*In vivo*	Inhibiting the process of fibrosis on nonalcoholic steatohepatitis in rats.	[97]
			Suppressing the development of hepatic steatosis, attenuating ultrastructural alterations of mitochondria of the hepatocyte, down-regulating the ROS in mice liver.	[98]
Siliphos	Fruits of *Silybum marianum (L.) Gaertn.*	*In vitro*	Alleviating the damage of lipid membranes by protecting of mitochondrial function.	[100]
		Clinical study	Decreasing ultrasonographic scores for liver steatosis in patients with nonalcoholic fatty liver disease.	[101]
Baicalin	Root of *Scutellaria baicalensis Georgi*	*In vivo*	Decreasing the level of serum total cholesterol, triglycerides, LDL, ALT and AST, and increase the level of serum HDL by mediation of CaMKKbeta/AMPK/ACC pathway.	[107]
		Clinical study	Attenuating the ischemia/reperfusion injury in alcoholic fatty liver by suppressing of myeloid differentiation factor 88 and TLR4 protein expressions and the nuclear translocation of NF-kB after reperfusion.	[108]
Gallic acid	Picarp of *Punica granatum L*	*In vivo*	Recovering impaired glucose and lipid homeostasis in high fat diet-induced NAFLD mice.	[95]
		*In vivo*	Reducing GSSG content and oxidative stress and increasing the levels of GSH peroxidase, glutathione, GSH S-transferase and GSH reductase in liver tissue.	[96]
Dioscin	Rhizoma of *Dioscorea opposita Thunb*	*In vitro* and *in vivo*	Relieving oxidative damage, inhibiting inflammation, cholesterol and triglyceride synthesis, decreasing MAPK phosphorylation levels, promoting fatty acid beta-oxidation, and inducing autophagy to improve fatty liver conditions.	[93]
		*In vivo*	Protective effect against alcoholic fatty liver by relieving alcohol-induced oxidative stress, inflammatory cytokine production, mitochondrial function, apoptosis and liver steatosis.	[94]
Total saponins of *Panax notoginseng (Burkill) F.H.Chen*	Flower, root, leaf of *Panax notoginseng (Burkill) F.H.Chen*	*In vitro*	Attenuating the ethanol induced hepatic lipid accumulation by inhibiting the production of MDA, GSH l and reactive ROS, reducing TNF-alpha and IL-6 levels, as well as enhancing the SOD)activity in liver, and abrogated CYP2E1 induction.	[88]
		*In vivo*	Relieve oxidative stress and insulin resistance in NAFLD rats.	
*Gynostemma pentaphyllum (Thunb.) Makino*	Aqueous extract of whole plant of *Gynostemma pentaphyllum (Thunb.) Makino*	*In vitro*	Promoting lipid metabolism, and decreasing serum lipids level by down-regulating the production of TMAO and up-regulating phosphatidylcholine.	[84]
			Inhibiting the accumulation of cholesterol and triglycerides as well as preventing oxidative stress by enhancing the production of NO and affects the molecular composition of the mitochondrial phospholipid CL.	[85]
		Clinical study	Reducing the level of serum AST, ALP, insulin, decrease BMI and insulin resistance index.	[86]
*Prunus armeniaca L*	Aqueous extract of kernels of *Prunus armeniaca L*	*In vivo*	Relieving CCl4-induced liver steatosis by antioxidant and radical-scavenging.	[103]
		Clinical study	Protective effects on fatty liver disease patient by decreasing the serum AST and ALT levels.	[104]

### 2.3. Herbs and Phytochemicals in the Treatment of Cirrhosis

#### 2.3.1. The Epidemiological and Pathological Characteristics of Cirrhosis and Current Therapeutic Strategy

Cirrhosis is a condition in which the liver does not function properly due to long-term damage; it is a terminal complication of chronic liver diseases. According to a study in Germany, the most common etiologies of liver cirrhosis in German were related to alcoholic liver disease (52%), chronic Hepatitis C (28%) or Hepatitis B (14%) infection and NASH (6%) [109]. However, the situation in developing countries is quite different; for example, in China, the distribution of etiological agents for cirrhosis was as follows: HBV 77.22%, alcohol 5.68%, HCV 2.80%, and the other etiologies accounted for about 15% [110]. Cirrhosis-related deaths increased from about 676,000 cases in 1980, or 1.54% of total global deaths, to more than 1,000,000 deaths in 2010, or 1.95% of the global deaths [111].

The pathological hallmark of cirrhosis is the development of fibrosis that replaces normal parenchyma. Fibrosis means overabound scar tissue builds up in the liver during wound healing and damage repair. The extracellular matrix (ECM) proteins such as collagens are excessively produced or deficiently degraded in this process, which may be due to the chronic inflammation or fatty liver. Fibrosis is potentially reversible if the cause is removed at this stage, but advanced fibrosis may further develop to cirrhosis which involves loss of hepatic cells and irreversible scarring in liver. Recent studies show the pivotal role of the stellate cells in the development of cirrhosis. Inflammation in the liver parenchyma can cause activation of hepatic stellate cells, which further leads to fibrosis formation and blood circulation obstruction. In addition, it secretes cytokines such as TGF-β1, which further promotes the fibrotic response and proliferation of connective tissue. Furthermore, it secretes tissue inhibitor of metalloproteinases (TIMPs) which inhibit the activity of matrix metalloproteinases (MMPs) and suppress them from resolving fibrotic material in the ECM [112]. Several mitogen-activated protein kinases regulate major fibrogenic actions of stellate cells. For example, c-Jun *N*-terminal kinase regulates apoptosis of hepatic cells and induces the secretion of inflammatory cytokines by stellate cell [113]. The focal adhesion kinase PI3K-Akt–signaling pathway regulates agonist-induced fibrogenic actions in stellate cell [114]. The TGF-β1–activated Smad-signaling pathway modulates experimental hepatic fibrosis *in vitro* and is a potential target for anti-fibrosis [115]. The PPAR pathway modulates stellate cell activation and fibrosis process. PPAR-γ ligands suppress the fibrogenic process in stellate cell *in vitro* and *in vivo* [116]. Recent studies suggest that NF-κB, Toll-like receptors and β-cathepsin may be involved in fibrosis regulation [117,118].

So far, there is no treatment that can radically reverse cirrhosis except for liver transplantation, but the supply of liver allografts is far short of the number of potential recipients [119]. Key prevention strategies for current cirrhosis management are through further prevention of liver damage by hepatitis or fatty liver disease. Unlike the irreversible process of cirrhosis, recent researches have proved that even advanced fibrosis is remediable. The appropriate anti-fibrosis therapy should target the reduction of lavish collagen deposition specifically without destroying normal ECM. Although no drugs have been approved for treating fibrosis or cirrhosis in USA, some herbal medicines have shown remarkable efficiency in preventing cirrhosis and treating fibrosis or relieving symptoms of cirrhosis (Table 3).

#### 2.3.2. Herbs and Phytochemicals in the Treatment of Cirrhosis

The hepatoprotective effect by CRAE on CCl_4_-induced chronic liver hepatotoxicity such as fibrosis has been reported by many studies [120,121]. These results showed that serum ALT and AST activities were remarkably decreased in CCl_4_-induced rats when treated with CRAE. The significant increase of serum SOD activity and the histological results proved that CRAE could recover CCl_4_-induced chronic oxidative stress by anti-oxidant mechanisms. In addition, another study found that the myofibroblast proliferation and the expression of TGF-b1 and a-SMA were remarkably decreased after berberine treatment [122]. These studies proved that CRAE and berberine could be promising anti-fibrosis agents in preventing and treating liver disease. The further mechanism study showed that the anti-fibrosis effects by berberine may be induced by activation of AMPK, blockade of Nox4 and Akt expression [123]. A comparative research on the hepatoprotective action of bear bile and CRAE on experimental liver fibrosis in rats demonstrated that berberine, CRAE, and bear bile all exhibited anti-fibrotic properties on liver fibrosis [124]. All of these agents could remarkably increase the SOD activity and reduce the peroxidative stress in rat liver. CRAE and berberine can protect hepatocytes from cholestatic damage through excreting bilirubin products from the liver. These results illustrated that CRAE and berberine may be promising agents to replace the valuable bear bile for liver fibrosis in clinical practice.

Puerarin is a kind of *isoflavones* isolated from *Pueraria lobata*. Recent studies have found that puerarin could promote the liver metabolic function and reduce the levels of ALT, AST and total-bilirubin, ECM contents and up-regulate the levels of albumin and total-protein in liver fibrosis rats. Further studies showed that puerarin could relieve the CCl_4_-induced liver fibrosis *in vivo*; the mechanisms may be related with modulation of the expression of PPAR-γ protein and PI3K/Akt pathway [125]. Another study found the TNF-alpha/NF-kB pathway was also involved in puerarin mediated anti-fibrosis effects in hepatic fibrotic rats. Through down-regulating the TNF-alpha and NF-kB expression by puerarin treatment, the inflammation response in liver tissue was relieved and the metabolic function was also improved [126]. Puerarin can also induce apoptosis of hepatic stellate cells, which plays a pivotal role in liver fibrosis. Further study of the mechanisms confirmed that down-regulation of bcl-2 mRNA are involved in puerarin induced hepatic stellate cells apoptosis [127].

*Saururus chinensis (Lour.) Baill* has been used in Chinese medicine for treating jaundice, gonorrhea and edema as well as reversing liver fibrosis [128,129]. *Saururus chinensis (Lour.) Baill* extract can effectively reduce the elevated levels of liver indexes such as serum ALT, AST, hyaluronic acid (HA), and hepatic MDA contents in hepatic fibrotic rats, it can also enhance the hepatic SOD activity after CCl_4_-treatment. The histopathological results proved that *Saururus chinensis (Lour.) Baill* extract significantly relieved the CCl_4_-induced liver fibrosis. These results demonstrated the hepatoprotective effect of *Saururus chinensis (Lour.) Baill* extract on CCl_4_-induced liver fibrosis [130].

The protective effect of glycyrrhizin on liver cirrhosis has been proved by both *in vitro* and *in vivo* studies. The mechanism of the anti-cirrhosis effect of glycyrrhizin perhaps has an inhibitory effect on NF-kB binding activity [131]. In this research, both the serum ALT detection and histological analysis results proved the anti-cirrhosis effect by glycyrrhizin. In addition, NF-kB binding activity was significantly decreased after glycyrrhizin treatment in the liver specimens of cirrhosis rats. 18alpha-glycyrrhizin, a modified derivative of glycyrrhizin, also showed obvious anti-fibrosis effects in CCl_4_-induced liver damage in rats. 18alpha-glycyrrhizin treatment improved histological changes and inhibited collagen deposition by decreasing the expressions of Smad2, Smad3, SP-1 and TGF-beta1 in both mRNA and protein level in the liver [132]. A phase III clinical study also confirmed the anti-fibrosis efficacy and safety of glycyrrhizin in 379 chronic Hepatitis C patients who failed to respond to interferon-based treatment. Results showed that 12-week treatment with 5×/week glycyrrhizin injections could decrease ALT level ≥50% in 28.7% patients and in the effect of 3×/week glycyrrhizin injections group the proportion was 29.0%, which were much more significant than placebo group (7.0%, *p* < 0.0001). Fifty-two week treatment can relieve the necro-inflammation in liver in 44.9% patients with 5×/week and in 46.0% patients with 3×/week, respectively. These results indicated that glycyrrhizin can reduce serum ALT level after 12 weeks of treatment and suppress necro-inflammation and fibrosis after 52 weeks of treatment compared to placebo group. In addition, no obvious side-effect by glycyrrhizin was detected in the clinical trial [133].

Silybinin, which is also known as *silybin*, exhibits antioxidant effect and induces mitochondrial biogenesis in cirrhotic livers [134]. Silybinin can prevent the production of mitochondrial reactive oxygen species (ROS) and inhibit the cardiolipin oxidation or citrate carrier failure in the liver of cirrhosis rat model. Silybinin also exhibits significant anti-inflammatory effect in cirrhotic rat liver by decreasing the expression of lysophosphatidylcholine acyltransferase (LPCAT) while increasing platelet-activating factor level [135]. A clinical survey indicated that silymarin can reduce symptoms and quality-of-life in cirrhosis patients. Long-term use of silymarin may provide benefits to patients with chronic Hepatitis C [136]. However, the effects of silymarin on survival rate or the disease progression of liver cirrhosis remains controversial in clinical practice [137].

*Bupleurum kaoi Liu*, *C.Y.Chao & Chuang*, a plant of endemic Bupleurum species in Taiwan, exhibits anti-fibrotic and anti-inflammatory activities in liver cells. These effects are proved to be associated with the anti-oxidant activity by observing the increased glutathione expression. It can also regulate the liver cell regeneration through the increased expression of IL-10 and INF-gamma. In addition, when compared with the *Bupleurum chinense DC*, the anti-fibrotic and hepatoprotective effects of *Bupleurum kaoi* are more significant [138]. Saikosaponin A, the major active ingredients in *Bupleurum chinense DC*, was found to increase the expression of bone morphogenetic protein 4 (BMP-4) and inhibit the activation of hepatic stellate cells [139]. When combining IFN-alpha treatment with saikosaponin, it could significantly reduce the damage of immune hepatic injury. In this study, the combination therapy can increase peripheral blood CD4+ T cell and CD8+ T cell ratios more significantly than using IFN-alpha alone. Meanwhile, the IL-18 and TNF-alpha levels both decreased obviously in combination therapy [140]. A recent study has indicated that saikosaponin A can be used for treatment of BMP-4 induced liver damage. Their result showed that the development of liver fibrosis was remised in the saikosaponin treatment group. The saikosaponin treatment can down-regulate plasma aspartate aminotransferase and alanine aminotransferase activities. In addition, the plasma and hepatic cholesterol and triglyceride levels also decreased after saikosaponin treatment [141].

The isolated Salvianolic acid B from *Salvia miltiorrhiza Bunge* has anti-fibrosis effect in liver diseases. Salvianolic acid B can inhibit intracellular signal transduction of TGF-β1 in hepatic stellate cells and suppress the expression of its receptor protein, thereby antagonizing the hepatic stellate cells activation, and inhibiting the fat-storing cell proliferation as well as reducing the deposition of collagen fiber in liver. A related animal study also confirmed that the *Salvia miltiorrhiza Bunge* treated liver tissues exhibited little fibrous tissue deposition in the portal areas, presented normal tissue morphology, and obviously decreased collagen deposition. In addition, decreased tissue inhibitor of metalloproteinase (TIMP)1 and collagen 1(alpha) protein also supports the anti-fibrotic effect of *Salvia miltiorrhiza Bunge* [142]. In animal studies, *Salvia miltiorrhiza Bunge* extracts have appeared to inhibit the process of liver fibrosis and improve liver function by reducing the nonfunctioning fibers in the liver. For people who have chronic hepatitis or excessive consumption of alcohol, *Salvia miltiorrhiza Bunge* is recommended for protecting their liver function [143].

The increase of glutathione S-transferase A5 expression and decrease of P450 cytochrome 3A2 by *Scutellaria baicalensis Georgi* extracts was also observed in liver cells. Treatment with methanolicextracts of *Scutellaria baicalensis Georgi* significantly reduced the levels of liver malondialdehyde and hydroxyproline with ameliorative histological results, which indicated the anti-fibrosis effect of *Scutellaria baicalensis Georgi* [144]. *Scutellaria baicalensis Georgi* extract also suppresses the proliferation and activation of hepatic stellate cells by inducing cell cycle arrest in G2/M phase and stellate cell apoptosis via caspases and Bax pathway [145]. Baicalein may be the main active component in *Scutellaria baicalensis Georgi* for prevention of cirrhosis and fibrosis. *In vivo* studies confirmed that baicalein can inhibit hypertrophic scar formation by suppressing TGF-beta/Smad2/3 signaling pathway in mice with mechanical load-induced scars [146]. Long-term administration of baicalein may suppress stellate cell activation by decreasing the expression of PDGF-beta receptor, and thus it can restrain the development process of liver fibrosis in animal models [147].

**Table 3:** Herbs and phytochemicals in cirrhosis treatment.

Herbal Medicines (Herbs or Phytochemicals)	The Sources of Herbal Medicines	Type of Study	Biological Effects and Molecular Mechanism	Reference
Berberine	Rhizoma of *Coptis chinensis Franch*	*In vivo*	Remedying CCl(4)-induced chronic oxide stress by anti-oxidant mechanisms, reducing myofibroblast proliferation and the expression of TGF- b1 and a-SMA.	[122]
Puerarin	Rhizoma of *Pueraria lobata*	*In vivo*	Attenuating the CCl4-induced toxicity in the hepatic cells of hepatic fibrosis rats, mediating anti-fibrosis effects through modulating the PPAR-gamma expression and inhibiting the PI3K/Akt signal pathway.	[125]
			Mediating anti-fibrosis effects in hepatic fibrosis rats through down-regulating the TNF-alpha and NF-kB expression.	[126]
			Inducing apoptosis of hepatic stellate cells by down-regulating bcl-2 mRNA.	[127]
Glycyrrhizin	Root of *Glycyrrhiza uralensis Fisch*	*In vivo*	Reducing serum ALT and improving histological changes by decreasing NF-kappa B binding activity.	[131]
			Improving histological changes and inhibited collagen deposition by decreasing the expressions of Smad2, Smad3, SP-1 and TGF-beta1.	[132]
		Clinical study	Reducing serum ALT level after 12 weeks of treatment and suppression of necro-inflammation and fibrosis.	[133]
Silybinin	Fruits of *Silybum marianum (L.) Gaertn.*	*In vivo*	Preventing the production of mitochondrial ROS and inhibiting the cardiolipin oxidation or citrate carrier failure in the liver of cirrhosis rat.	[134]
			Exhibiting significant anti-inflammatory effects in cirrhotic rat liver by decreasing the expression of LPCAT and increasing platelet-activating factor level.	[135]
Saikosaponin A	Root of *Bupleurum kaoi Liu*, *C.Y.Chao & Chuang*	*In vitro*	Increasing the expression of BMP-4 and inhibiting the activation of hepatic stellate cells.	[139]
		*In vivo*	Combination therapy with IFN-alpha can increase peripheral blood CD4+ T cells and CD8+ T cell ratios, down-regulating plasma AST and ALT activities, decreasing the plasma and hepatic cholesterol and triglyceride levels.	[140,141]
Salvianolic acid B	Root and rhizome of *Salvia miltiorrhiza Bunge*	*In vitro*	Inhibiting intracellular signal transduction of TGF-β1 in hepatic stellate cells and suppressing the expression of its receptor protein.	[142]
		*In vivo*	Decreasing fibrous tissue deposition in the portal areas, and obviously decreasing collagen deposition. In addition, decreasing oTIMP1 and collagen 1(alpha) protein.	
			Inhibiting the process of liver fibrosis and improve liver function by reducing the nonfunctioning fibers in the liver.	[143]
Baicalein	Root of *Scutellaria baicalensis Georgi*	*In vivo*	Inhibiting hypertrophic scar formation by suppressing TGF-beta/Smad2/3 signaling pathway in mice with mechanical load-induced scars.	[146]
			Suppressing stellate cell activation by decreasing the expression of PDGF-beta receptor.	[147]
Silymarin	Flavonolignans mixture from the fruits of *Silybum marianum (L.) Gaertn.*	Clinical study	Long-term use may reduce symptoms and quality-of-life in cirrhosis patients.	[137]
*Coptis chinensis Franch*	Aqueous extract of rhizoma of *Coptis chinensis Franch*	*In vivo*	Increasing serum SOD activity and the histological results in CCl(4)-induced liver fibrosis by anti-oxidant mechanisms.	[120,121]
*Saururus chinensis (Lour.) Baill*	Ethanol extract of rhizoma of *Saururus chinensis (Lour.) Baill*	*In vivo*	Reducing the elevated levels of liver index such as serum ALT, AST, HA, and hepatic MDA contents in hepatic fibrosis rats.	[130]
*Bupleurum kaoi Liu*, *C.Y.Chao & Chuang*	Ethanol extract of root of *Bupleurum kaoi Liu*, *C.Y.Chao & Chuang*	*In vitro*	Exhibiting the anti-fibrotic and anti-inflammatory activities in liver cells by anti-oxidant activity of increasing glutathione expression.	[138]
*Scutellaria baicalensis Georgi*	Ethanol extract of root of *Scutellaria baicalensis Georgi*	*In vitro*	Reducing the levels of liver malondialdehyde and hydroxyproline significantly, with ameliorative histological results which indicated the anti-fibrosis effect.	[144]
			Suppressing the proliferation and activation of hepatic stellate cells by inducing cell cycle arrest in G2/M phase and inducing stellate cell apoptosis via caspase and Bax pathway.	[145]

### 2.4. Herbs and Phytochemicals in the Treatment of Primary Liver Cancer

#### 2.4.1. The Epidemiological and Pathological Characteristics of Primary Liver Cancer and Current Therapeutic Strategy

The incidence of primary liver cancer is increasing in many industrialized countries, it is the sixth most frequent diagnosed cancer globally, and has become the second leading cause of cancer death since 2014. In 2010, primary liver cancer become the third leading cause of cancer death worldwide, with 754,000 deaths related with this disease. In 2012, about 782,000 people were diagnosed with primary liver cancer and 746,000 people eventually died from this disease. The occurrence of liver cancer may be associated with hepatitis virus infection (71%) and alcoholic abuse (20%). In 2014, liver cancer resulted in more than 750,000 deaths globally [148].The incidence of liver cancer is higher in those countries where hepatitis virus infection are common, such as East-Asia and sub-Saharan Africa [149]. The five year survival rate of primary liver cancer is only 17% in the United States in 2014 and even lower in developing countries [150]. Hepatocellular carcinoma (HCC) is the most common type of primary liver cancer, which shows an obvious geographical distribution. For example, 50% of HCC are diagnosed in China [151].

The leading cause of liver cancer is cirrhosis induced by viral hepatitis or fatty liver disease. Hepatitis or fatty liver promote the development of HCC through repeatedly inducing the immune system to attack the hepatic cells, while this constant cycle of damage followed by repair can lead to mistakes during repair which in turn lead to carcinogenesis. For chronic Hepatitis B, the integration of the viral genome into the host liver cells can directly induce gene mutation and thereby develop HCC. The disorders in Wnt, TGF-β, Hedgehog and Notch signaling pathways play a pivotal role in the oncogenesis process of HCC. Some other factors such as microRNAs may also regulate the liver oncogenesis process [152,153].

So far, the treatment for liver cancer is limited, the later stage or higher-grade liver cancer patients will ordinarily have poor prognosis [154]. Surgical resection may be the best therapy for promoting long-term survival, but because most patients are diagnosed at the later stage of disease, therefore only 10% of the patients are suitable for surgical resection. Sorafenib, a receptor tyrosine kinase inhibitor which has been approved in the US and Europe, may be beneficial for patients with advanced HCC. Unfortunately, Sorafenib increases lifespan by only two months for late stage HCC patients with satisfactory liver function according to a phase III clinical trial [155]. Under these circumstances, herbal medicines are recommended as an alternative treatment for liver cancer patients, in order to reduce the side-effects from conventional therapy and prolong survival time as well as improve life quality (Table 4).

#### 2.4.2. Herbs and Phytochemicals in the Treatment of Primary Liver Cancer

The recent pharmacological studies have demonstrated that both *Coptidis rhizoma* and berberine have a prominent hepatoprotective effect, especially the anti-cancer effect on hepatocellular carcinoma. A recent research has shown that the alkaloids in *Coptidis rhizoma* aqueous extract (CRAE) inhibited vascular endothelial growth factor (VEGF) by suppressing the activity of eukaryotic elongation factor 2 (eEF2) in liver cancer [156]. The phytochemical study showed that the major alkaloids in CRAE are berberine, jatrorrhizine, magnoflorine and palmatine. These alkaloids could suppress VEGF secretion in HepG2 and MHCC97L cells while no significant toxicity has been observed in normal liver cells. This inhibition effect was mainly observed for protein level rather than mRNA transcriptional level. The alkaloids in CRAE also inhibited the activation of eEF2 by raising the phosphorylation levels of eEF2 and thereby blocked the nascent protein synthesis. Berberine was found to be the main active ingredient in these alkaloids but the total alkaloids in CRAE is more effective in inhibiting the activity of eEF2. The *in vivo* study also confirmed that the tumor size of xenograft mice was significantly reduced after *Coptidis rhizoma* treatment. According to another study by our research group, miR-23a expression can be upregulated by berberine treatment in human HCC cells and may activate the transcription of p53-related tumor suppressive GADD45alpha and p21 genes. *In vivo* study also showed that when miR-23a expression was inhibited, the berberine-induced tumor suppression in mice was attenuated [157]. Another study also confirmed that CRAE suppressed HCC growth by targeting on miR-21 and miR-23a [158]. The invasion-chamber and wound-healing experiments showed that CRAE could suppress HCC migration through inhibition of Rho/ROCK signaling pathway and the total alkaloids in CRAE may be promising agents for preventing cancer invasion [159]. On the other hand, we also found that berberine could induce HCC cell death via apoptosis and autophagy. The mechanisms may be related with activation of Beclin-1 to induce mitochondrial apoptosis and suppression of mTOR to induce autophagic cell death in HCC by berberine [160].

As complementary herbal medicine, long term administration of silymarin can inhibit cell proliferation, induce cell apoptosis, and sensitize the detoxification system in HCC cells [161]. The chemopreventive effect of silymarin on HCC has been demonstrated in both *in vitro* and *in vivo* studies [162]. It can interfere with cytokines’ expression and regulate cell survival and apoptosis. Moreover, silymarin can inhibit inflammation and metastasis of HCC. The potential molecular mechanisms are associated with reduced mitochondrial transmembrane potential of liver cells followed by increased expression of cytosolic cytochrome c and pro-apoptotic proteins such as Bax, p53, caspase-3 and APAF-1 and down-regulation of anti-apoptotic proteins (survivin and Bcl-2) and proliferation-related proteins (beta-catenin, c-Myc, PCNA, and cyclin D1) [163]. All these further suggested that silymarin could be a promising adjuvant agent for HCC treatment.

When combining *Bupleurum chinense DC* with chemotherapy, increased micronuclei frequency and DNA damage have been observed on liver cancer cells [164]. When saikosaponin D was used to treat HepG2 cells, a significant increase of cell apoptosis was observed. The mechanisms of apoptosis may associate with the activation of caspases 3 and 7 and finally cause DNA fragmentation. Saikosaponin D also sensitizes radiotherapy on hepatoma cells under hypoxic conditions by suppressing HIF-1 alpha expression [165]. *In vivo* studies found that saikosaponin D can inhibit HCC development and metastasis by decreasing the expression of syndecan-2, MMP-2, MMP-13 in rat liver cells [166].

Rui *et al.* found that *Salvia miltiorrhiza Bunge* extracts prevents human HCC on DEN-induced hepatocarcinogenesis by suppressing fibrosis and plasminogen activator inhibitor-1 mRNA transcription in a dose-dependent manner [167]. Further studies found that tanshinones may be the major anti-cancer ingredient in *Salvia miltiorrhiza Bunge* with the observation in *in vitro* studies that significant cytotoxic effects were incurred by tanshinones on doxorubicin-resistant human HCC cells. The cryptotanshinone suppressed doxorubicin efflux, a process mediated by P-glycoprotein, in Pgp-overexpressed drug resistance HepG2 cells. The tanshinone IIA, another kind of tanshinone, exhibits pro-apoptotic effects and better synergism with doxorubicin in HCC cells [168]. Another study also confirmed the cytotoxicity of tanshinone IIA in HCC cells. Tanshinone IIA can induce cell apoptosis without influencing oxidative stress *in vitro*. However, other tanshinones in the study have not shown significant effect in inducing apoptosis in HCC cells [169].

The anti-cancer and anti-metastatic effect of wogonin have been reported in many studies. The actions of wogonin may be attributed to suppression of VEGF C-induced lymphangiogenesis as postulated by the decrease in VEGF-C-induced VEGFR-3 phosphorylation through suppression of IL-1beta and COX-2 [170]. Pretreatment with wogonin also obviously down-regulated hepatic DNA adducts’ formation in mice which indicated that wogonin might have protective effects against genotoxicities [171]. Although the anti-cancer effects of wogonin has been proved in many *in vitro* studies, the *in vivo* application of wogonin is limited due to its poor solubility and lack of specificity. Study on the anti-HCC effect by wogonin showed that modified wogonin liposomes can improve the bio-distribution, accumulation of the drug at the tumor tissue and enhance the therapeutic efficacy on liver cancer [172].

Elemenes are a group of closely related natural compounds found in *Curcuma aromatica Salisb*. Recent study found that beta-elemene can induce cell apoptosis and suppress the proliferation of HepG2 cells, and the mechanism may be related to the suppression of microtubular polymerization and decrease of alpha-tubulin [173]. Bao *et al.* found that beta-elemene can suppress the growth of H22 ascites hepatoma cells in a dose-and time-dependent manner. According to the study, beta-elemene can suppress the growth of H22 cells through increasing the expression of histone H1 protein. These findings indicated that beta-elemene may be a potential antitumor agent for liver cancer treatment [174].

*Brucea javanica (L.) Merr* extracts have been found to have low toxicity but potent anti-cancer effect in many studies. Recent studies have found that *Brucea javanica (L.) Merr* extracts can induce liver cancer cell apoptosis by regulating mitochondrial dependent pathway and activating caspase 3 [175]. Yue *et al.* demonstrated that *Brucea javanica (L.) Merr* extracts can significantly suppress the proliferation of HepG2 cells in a dose-dependent manner. Compared with the control group, the *in vivo* treatment group of *Brucea javanica (L.) Merr* extracts had lower metastasis rate and higher body weight. The *Brucea javanica (L.) Merr* extracts induce apoptosis in hepatocellular carcinoma tissue in a dose-dependent manner [176].

Camptothecin, a cytotoxic quinoline alkaloid, is widely used as DNA enzyme topoisomerase I inhibitor in cancer treatment. It was isolated from the bark or stem of *Camptotheca acuminata Decne*, a tree distributed in China and has been used as an anti-cancer herb in traditional Chinese medicine. Li *et al.* found that camptothecin can suppress SMMC-7721 cell growth by arresting cell cycle at the S and G2/M phases, and induce mitochondrial pathway mediated apoptosis *in vitro* [177]. Camptothecin can also induce TRAIL-mediated apoptosis in HCC cells by increasing ROS and ERK/p38-dependent DR5. Further studies confirmed that pretreatment of HCC cells with antioxidants or DR5 specific inhibitors of p38 and ERK can decrease camptothecin-TRAIL-induced cell apoptosis by inhibiting DR5 expression [178]. *In vivo* studies proved that the encapsulated camptothecin with *N*-trimethyl chitosan (CPT-TMC) may have high solubility and better efficacy for liver cancer treatment. In hepatic carcinoma bearing BALB/c mice model, CPT-TMC significantly suppressed tumor growth and lymphatic metastasis as well as extended survival time, while no obvious side-effect is observed. Thus, CPT-TMC may be a potential safe and effective therapeutic agent for liver cancer treatment [179].

Curcumin is a diarylheptanoid isolated from turmeric which belongs to the ginger family. It is originally used as food additive in food coloring, and it has also been investigated in many studies for its remarkable *in vitro* and *in vivo* anticancer effects. Notarbartolo *et al.* found that when curcumin used alone or in combination with doxorubicin or cisplatin can inhibit cell proliferation and induce apoptosis in human liver carcinoma cells. The researchers further explored the changes in IAP gene expression and NF-kB activation levels after curcumin treatment. However, there are no significant changes in NF-kB activation, which indicated the anti-cancer effects by curcumin may be related to other mechanisms [180]. For curcumin-resistant liver cancer cells, a recent study has revealed that Chk1-mediated G2/M cell cycle arrest may be related with curcumin resistance and Chk1 may be a potential target for future study in reversing resistance and improving the clinical efficacy of curcumin [181].

**Table 4:** Herbs and phytochemtableicals in primary liver cancer treatment.

Herbal Medicines (Herbs or Phytochemicals)	The Sources of Herbal Medicines	Type of Study	Biological Effects and Molecular Mechanism	Reference
Berberine	Rhizoma of *Coptis chinensis Franch*	*In vitro* and *in vivo*	Increasing miR-23a expression in human HCC cells and may activate the transcription of p53-related tumor suppressive GADD45alpha and p21 genes.	[158]
		*In vitro*	Inducing mitochondrial apoptosis in liver cancer cells and activating autophagic cell death in liver cancer cells by activation of Beclin-1 and suppressing the mTOR-signaling pathway.	[160]
Saikosaponin D	Root or leaf of *Bupleurum chinense DC*	*In vitro*	Increasing cell apoptosis by activation of caspases 3 and 7 and finally causing the DNA fragmentation.	[165]
		*In vitro*	Inhibiting HCC development and metastasis by decreasing the expression of syndecan-2, MMP-2, MMP-13 in rat liver cell.	[166]
Cryptotanshinone, Tanshinone IIA	Root and rhizome of *Salvia miltiorrhiza Bunge*	*In vitro*	Suppressing doxorubicin efflux by regulating P-glycoprotein expression.	[168]
		*In vitro*	Inducing apoptotic effects without influencing oxidative stress and synergism with doxorubicin in HCC cells.	[168]
Wogonin	Root of *Scutellaria baicalensis Georgi*	*In vitro*	Suppressing the VEGF-C-induced lymphangiogenesis by a decrease in VEGF-C-induced VEGFR-3 phosphorylation through suppressing of IL-1beta and COX-2 production.	[170]
		*In vivo*	Exhibiting protective effects against genotoxicities by down-regulating hepatic DNA adducts’ formation in mice.	[171]
Beta-elemene	Rhizome of *Curcuma aromatica Salisb*	*In vitro*	Inducing cell apoptosis and suppressing the proliferation of HepG2 cells by suppressing microtubular polymerization and decreasing alpha-tubulin.	[173]
		*In vitro*	Suppressing the growth of H22 cells by increasing the expression of histone H1 at the protein level.	[174]
Camptothecin	Bark or stem of *Camptotheca acuminata Decne*	*In vitro*	Suppressing SMMC-7721 cell growth by arresting cell cycle at the S and G2/M phases, and inducing mitochondrial pathway mediated apoptosis.	[177]
		*In vitro*	Inducing TRAIL-mediated apoptosis in HCC cells by increasing ROS and ERK/p38-dependent DR5.	[178]
Curcumin	Rhizome of *Curcuma longa L*	*In vitro*	Inhibiting cell proliferation and inducing apoptosis on human liver carcinoma cells.	[180]
Silymarin	Flavonolignans mixture from the fruits of *Silybum marianum (L.) Gaertn.*	*In vivo*	Inhibiting cell proliferation, inducing cell apoptosis, and sensitizing the detoxification system in hepatocellular carcinoma cells.	[161]
		*In vitro* and *in vivo*	Inhibiting inflammatory activity and metastases of HCC cells by down-regulating the mitochondrial transmembrane potential of liver cells.	[163]
*Coptidis rhizoma*	Aqueous extract of rhizoma of *Coptis chinensis Franch*	*In vitro* and *in vivo*	Inhibiting VEGF by suppressing the activity of eEF2 in liver cancer	[156]
		*In vitro*	Suppressing HCC cells migration through Rho/ROCK signaling pathway inhibition.	[159]
*Bupleurum chinense DC*	Aqueous extract of root or leaf of *Bupleurum chinense DC*	*In vitro*	Increasing the micronuclei frequency and DNA damage in liver cancer cells in combination with chemotherapy.	[164]
*Salvia miltiorrhiza Bunge*	Aqueous extract of root and rhizome of *Salvia miltiorrhiza Bunge*	*In vivo*	Preventing human HCC on DEN-induced hepatocarcinogenesis by suppressing fibrosis and plasminogen activator inhibitor-1 mRNA transcription.	[167]
*Brucea javanica (L.) Merr*	Aqueous extract of seeds of *Brucea javanica (L.) Merr*	*In vitro*	Inducing liver cancer cell apoptosis by regulating the mitochondrial dependent pathway and activating caspase 3.	[175]
		*In vitro* and *in vivo*	Suppressing the proliferation of HepG2 cells in a dose-dependent manner and inhibiting metastasis.	[176]

## 3. Traditional Chinese Medicine Herbal Formulae for Chronic Liver Disease Treatment

**Figure 1:**
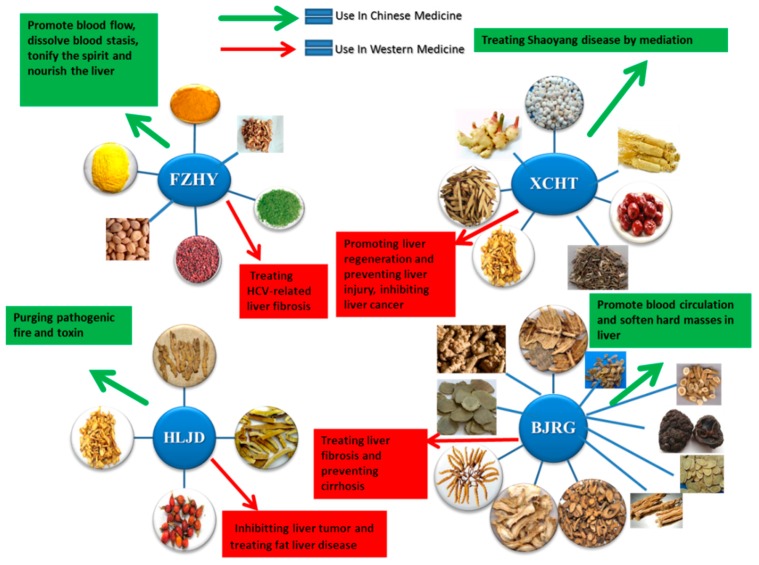
The traditional Chinese medicine (TCM) herbal formulas for chronic liver disease treatment. FZHY = Fuzheng Huayu tablet; XCHT = Xiao Chai Hu Tang; HLJD = Huanglian Jiedu decoction; BJRG = Biejia ruangan tablet.

With the rapid development of Omicsresearch such as genomics, proteomics and metabolomics, the study of the essence of traditional Chinese medicine (TCM) herbal formulas in clinical therapy has received more attention worldwide [182,183]. Different from Western medicine which focuses on specific pathogenic processes, TCM emphasizes the importance of individualized therapy based on syndrome differentiation. The TCM herbal formulas which consist of several herbs, mineral or animal organs have been applied in treating liver diseases for thousands of years in China and some Asian countries (Figure 1). Although some studies including clinical trials have proved that TCM herbal formula have multiple targets in treating liver disease [184,185,186], the related mechanisms of the hepatoprotective effects of these herbal formulas are still not fully understood.

### 3.1. Fuzheng Huayu Tablet for Chronic Liver Disease Treatment

Fuzheng Huayu (FZHY) tablet was the first TCM formula which completed a US Food and Drug Administration (FDA) phase II clinical trial for liver disease treatment. The FZHY formula consists of six types of traditional Chinese medicines: *Semen persicae*, *Fructus Schisandrae chinensis*, *Radix Fermentation Mycelium Powder*, *Salvia miltiorrhiza Bungee*, *Gynostemma pentaphyllammak* and *Pollen Pini* [187]. According to a double-blinded, placebo-controlled, multi-center clinical phase II trial, FZHY tablet could be an effective and safe treatment for liver fibrosis in chronic Hepatitis C patients. The efficacy of FZHY treatment was confirmed by statistical analysis using Bayes classification model. After FZHY treatment, the levels of both hyaluronic acid and TCM syndrome score were significantly decreased (*p* < 0.05) compared to placebo group [188]. A recent study showed that the FZHY formula prevents fibrosis and nourishes steatohepatitis by regulating the oxidative stress related genes and the IKKbeta/NF-kB, and TGF-beta1/Smad signaling pathways in mice model [189]. For HBV related liver damage, FZHY recipe also showed good therapeutic effects in down-regulating the levels of AST, ALT as well as HBV DNA concentration. A recent clinical trial study also showed that FZHY capsule significantly decreases the ratio of TGF-beta1/BMP-7 mRNA in chronic viral Hepatitis B related fibrosis patients. FZHY could significantly decrease the ratio of TGF-beta1/BMP-7 (0.09 *vs.* 0.25, *p* < 0.05). In terms of pathological changes of the liver, the inflammatory activity for G3 and fibrosis degree S3 in the FZHY group was significantly lower than that in the control group (*p* < 0.05). In addition, the drug resistance related HBV YMDD variation in control group was higher than in the FZHY group [190]. In a multicenter clinical study, FZHY capsule showed satisfactory effects in treating chronic Hepatitis B related liver fibrosis with fewer adverse effects. Compared to another traditional hepatoprotective formula, the Heluoshugan capsule, the FZHY capsule had a better effect in suppressing inflammatory activity [191]. The FZHY tablet could inhibit the autocrine activation pathway of hepatic stellate cells. This effect may be associated with the suppression of VEGF in hepatic stellate cells [192]. As one of the patented TCM formulas, the phase III studies of FZHY tablet has just started in the US in 2014, which will give more information on the safety and efficacy of the FZHY tablet in different populations in the future.

### 3.2. Xiao Chai Hu Tang for Chronic Liver Disease Treatment

Xiao Chai Hu Tang (XCHT), a TCM herbal formula originally recorded in the ancient Chinese classic book ’Shang-Han-Lun’, has been used to treat chronic liver disease for more than 2000 years in China and some Asian countries, such as Japan where it has been approved as an ethical drug [193]. The main ingredients in this formula include *Bupleurum*, *Scutellaria*, *ginseng*, *licorice*, *pinellia*, *ginger* and *jujube*. As one of the most popular TCM formulas, there are many studies showing that CHXT can improve liver function and promote liver regeneration as well as prevent liver damage. According to previous research, CHXT has been used to treat hepatitis, liver fibrosis and even hepatocellular carcinoma. There are currently ongoing clinical trials for anti-Hepatitis C effect by XCHT at the University of California, San Diego and Memorial Sloan-Kettering Cancer Center. Another research group in Singapore also found that XCHT could inhibit the production of HBV, but the major ingredients contributing to the activity are still not clear [194]. The previous study with children as the patient group with HBV infection also showed improvement on HBeAg clearance rate by SCHT [195]. In addition to the anti-HBV effect, another clinical trial showed that XCHT could improve liver pathology in Hepatitis C patients. The results demonstrated that XCHT could down-regulate the expression of AST and ALT in most of the Hepatitis C patients. Decrease of AST levels was observed in 16/24 patients. Decrease of ALT levels was seen in 18/24 patients. Seven patients showed decrease in viral load while 10 patients showed increase, and the results of the other seven patients were indeterminate because of assay limitations. Moreover, the subsequent liver biopsy histology also supported the hepatoprotective effect of XCHT in HCV patients [196]. To study the anti-fibrosis effect of XCHT, one group used the bile duct ligation rats to evaluate the anti-fibrotic potential by this herbal formula. Their results showed that XCHT could decrease cholestasis in bile duct ligation rats and significantly down-regulate the collagen content. More importantly, XCHT exhibited remarkable antifibrogenic effect through decreasing the expression of TIMP-1 and procollagen alpha1 types (I) and (III) in liver [197]. Further studies by the same group demonstrated that XCHT could decrease the serum fibrotic marker PIII NP in bile duct ligated rats through decreasing the expression of platelet-derived growth factor and the transforming growth factor beta1. At the same time, XCHT could induce the expression of TNF-alpha to suppress hepatic stellate cell proliferation and collagen formation [198]. The role of SCHT in liver cancer is still controversial. Some studies found that XCHT could prevent tumor formation in patients with hepatic cirrhosis, especially in those without HBV infection [199]. However, another study demonstrated that 70-week administration of XCHT could not prevent tumorigenesis in rats with spontaneous hepatocarcinogenesis [200].

### 3.3. Huanglian Jiedu Decoction in Liver Disease Treatment

The Huanglian Jiedu (HLJD) decoction originally appeared in “Essentials from the Imperial Library” (Wai Tai Bi Yao) in DC 752. According to the basic theory of TCM, it is used for “Clearing Heat from the Qi Level”. In Japan, HLJD decoction has been approved as a conventional drug for alcoholic and drug-induced liver disease. There are four substances in this TCM formula: *Rhizoma Coptidis*, *Scutellariae Baicalensis*, *Cortex Phellodendri* and *Fructus Gardeniae*. Our recent findings showed that HLJD decoction can inhibit liver tumor growth by suppressing the expression of eukaryotic elongation factor-2(eEF2) in HCC cells. HLJD decoction could suppress HCC cell proliferation without significant side-effects. *In vivo* study showed that it could inhibit tumor growth and angiogenesis in xenografted mice. Further study of the mechanisms demonstrated that HLJD decoction could inhibit the expression of nascent protein by suppressing the activation of eEF2 in liver cells. The major active ingredients in HLJD decoction, geniposide, baicalin and berberine may synergistically activate eEF2 kinase (eEF2K) and lead to eEF2 inactivation [201]. Similar results presented by another group also confirmed that HLJD decoction was effective in other cancers [202]. The protective effects of HLJD decoction in fatty liver disease in hyperlipidemia mice model might be mediated by damaged tissue repair and immune-regulation of M2 macrophage subpopulations [203]. Another study of hyperlipidemia rats showed different anti-steatosis mechanisms of HLJD and confirmed that the activation of lipid metabolism enzyme and the increased expressions of LDLR and PPAR-gamma are important in preventing liver steatosis in the rats [204].

### 3.4. Biejia Ruangan Tablets in Liver Disease Treatment

According to TCM theory, the Biejia Ruangan (BJRG) tablets can promote blood circulation and soften hard masses in liver. The components of BJRG tablets include nine kinds of herbs (*Rhizoma curcumae*, *Radix Paeoniae rubra*, *Radix Angelica sinensis*, *Radix notoginseng*, *Radix codonopsis*, *Astragalus membranaceus (Fisch.) Bunge*, *Placenta hominis*, *Radix isatidis*, *Fructus forsythia*), an animal tissue (turtle shell) and a complex of fungus and larva (*Ophiocordyceps sinensis*). BJRG tablets were the first Chinese FDA approved herb compound for treatment of liver fibrosis. Over the past decades, the role of BJRG tablet in prevention and treatment of fibrotic liver disease has been verified by many experiments and clinical trials. BJRG tablet could inhibit the development of fibrosis by decreasing the mRNA expressions of connective tissue growth factor in rats [205]. Another study using renal cells also confirmed the anti-fibrosis effects of BJRG tablet and the mechanism may be related with its suppression on TIMP-1 and type I and III procollagen expression [206]. In the rat liver fibrosis model induced by carbon tetrachloride, there is a reduction in liver collagen deposition and improvement in hepatic injury. The early cirrhosis related serum indicators such as PCIII, HA, laminin (LN) and collagen IV (CIV) were obviously decreased. Further mechanism study revealed that the suppression of TGF-beta1 and Smad3 expression may contribute to the hepatoprotective effects of BJRG tables [207]. A clinical trial with 420 patients of HBV-related liver fibrosis found that 500 mg/day BJRG tablet may alleviate clinical symptoms and hepatic fibrosis (55.67 percent and 81.67 percent *vs.* 15.8 percent and 60.00 percent, *p* < 0.01) compared with the control group. Also, there is no obvious adverse effects detected during the treatment [208]. In addition, another study found that increased dose of BJRG tablets to 1000 mg/day showed a larger pharmacological and pharmacokinetic area under the curves (AUCs) and better therapeutic effects than 500 mg/day or 1500 mg/day administration of BJRG tablet [209].

## 4. The Potential Side-Effects or Toxicity of Herbal Products in Chronic Liver Disease Treatment

Although most people consider herbal products as natural and safe agents, there have been some clinical case-reports about side-effects or toxicity of herbal products in recent years. Some side effects or toxicity of herbal medicines may constitute an acute exacerbation such as allergy, while some agents may have chronic toxicity which require long-term accumulation such as some nephrotoxicity herbs [210,211]. Herein, we reviewed the side-effects and toxicity of several conventional herbal products in liver disease treatment.

### 4.1. The Potential Side-Effects or Toxicity of Coptis Chinensis Franch and Berberine in Chronic Liver Disease Treatment

The acute toxicity study in mice demonstrated that LD50 of *Coptis chinensis Franch* was approximately 4.9 g/kg by oral and the LD50 values of berberine by intravenous injection and intraperitoneal injection has been proved to be 9.0386 and 57.6103 mg/kg [212,213]. The most common side effects of berberine include laxative, constipation, anaphylaxis and some skin allergies. One study suggested that berberine may cause the degeneration of dopaminergic neuronal cells in Parkinson’s disease rat model with long-term levodopa treatment. Berberine administration should be monitored for Parkinson's disease patients with chronic L-DOPA treatment [214]. Some other studies believed that berberine treatment for pregnant women may lead to hemolytic disease in newborns; berberine treatment for children may lead to severe jaundice and acute hemolysis [215,216,217,218]. Prescriptions of berberine have been prohibited in some countries such as the USA and Singapore, but it is still used legally in China (including Hong Kong) in clinical practice.

### 4.2. The Potential Side-Effects or Toxicity of Glycyrrhizin in Chronic Liver Disease Treatment

Previous study has proved that glycyrrhizin can lead to pseudoaldosteronism which will cause aldosteronopenia, hypokalemia, hypertension, and inhibited plasma renin activity [219]. Since *Glycyrrhiza uralensis Fisch* raised the blood pressure in dose-related manner, previous study has proved that consumption of 50 g of *Glycyrrhiza uralensis Fisch* (containing 75 mg glycyrrhetinic acid) per day for two weeks can significantly raise the blood pressure. These results have important implications for hypertension patients consuming *Glycyrrhiza uralensis Fisch*. Both *in vitro* and *in vivo* studies of the licorice extracts exhibit estrogen-like activities which may lead to a high risk of heart disease in women [220]. Another study also found that high dose administration of glycyrrhizin for pregnant women will increase the premature birth rate [221]. For male patients, licorice extracts can lead to down-regulation of serum testosterone secretion and cause impotency [222]. Other studies have confirmed that high doses of glycyrrhizin can induce hypermineralocorticoid-like effects. Based on the clinical evidence, the daily intake of glycyrrhizin should be lower than 0.2 mg/kg [223].

### 4.3. The Potential Side-Effects or Toxicity of Silymarine and Silybinin in Liver Disease Treatment

A phase I clinical trial in prostate cancer patients demonstrated that high dose silibinin (13 g/day) may cause hyperbilirubinemia and increase alanine aminotransferase levels [224]. Other side effects include regulation of endocrine functions via blockade of estrogen receptor and aryl hydrocarbon receptor (AhR) has also been reported. The estrogenic effects by silymarine should also be paid attention to. An *in vivo* experiment on ovariectomized rats demonstrated that silymarine can obviously inhibit the bone loss in ovariectomized rats with mild proliferative effects in uterus. Generally, silymarine and silybinin have not shown any significant side effects or toxicity in previous study, except for the above mentioned ones [225,226,227].

### 4.4. The Potential Side-Effects or Toxicity of Bupleurum Chinensis DC and Saikosaponins in Chronic Liver Disease Treatment

Since the toxicity incidences of Xiao Chai Hu Tang have been reported in Japan, the side effects and toxicity of Bupleurum have been the focus of much attention. Interstitial pneumonia can be induced by combination treatment of interferon with Xiao Chai Hu Tang, and Bupleuri was believed to be the key component in the decoction [228]. The single oral dose of Bupleuri in mice showed that the LD50 of Bupleurum extracts was over 2000 mg/kg [229]. The most common side effects of Bupleurum include increased bowel movements, intestinal gas, and drowsiness. Bupleurum could excessively activate the immune system and might increase the symptoms of auto-immune diseases [230]. Alcohol eluent of saikosaponins can induce liver damage by inhibiting the respiratory chain system of mitochondria and suppressing liver energy metabolism [231]. Another study demonstrated that the consumption of crude extracts of total saikosaponins may lead to liver damage, and the mechanism of this damage may be attributed to oxidative damage [232]. Saikosaponins D also reduce the cell viability and cause dramatic morphological changes in liver L02 cells. However, some may argue that the incorrect use of Bupleuri may be the main cause of the adverse effects. The safe use of Bupleuri still needs further investigation [233,234].

### 4.5. The Potential Side-Effects or Toxicity of Salvia Miltiorrhiza Bunge in Chronic Liver Disease Treatment

In 2010, the compound Danshen dripping pills became the first Chinese herbal medicine which successfully completed US FDA Phase II clinical trials. As a major component of *Salvia miltiorrhiza Bunge*, it should constitute a promising, safe medicinal plant for liver disease treatment. However, there are still some side effects which should be paid serious attention to in clinical practice. *Salvia miltiorrhiza Bunge* can inhibit hemostasis through suppressing platelet aggregation, enhancing fibrinolytic activity, and inhibiting the extrinsic blood coagulation. At the same time, it could enhance the anticoagulant effect by warfarin. Thus, for patients taking warfarin, *Salvia miltiorrhiza Bunge* treatment should be avoided to decrease the risk of bleeding complications [235,236,237]. Some clinical research also found that *Salvia miltiorrhiza Bunge* injection may lead to anaphylactic reaction on animal models, while negative reaction was observed when treated with the liquid excipients of *Salvia miltiorrhiza Bunge* injection [238]. This may be attributed to some antigenic impurities which could not be removed completely in the preparation process of *Salvia miltiorrhiza Bunge* injection. Other possible side effects include reduced appetite, convulsions or itching. Some people may experience mental changes, stomach discomfort and dystonia syndrome after *Salvia miltiorrhiza Bunge* treatment.

### 4.6. The Potential Side-Effects or Toxicity of Scutellaria Baicalensis Georgi in Chronic Liver Disease Treatment

Usually, treatment with *Scutellaria baicalensis Georgi* will not cause serious side effects. Occasionally, it may induce lung inflammation, fever, or drowsiness. Chronic administration of *Scutellaria baicalensis Georgi* may cause hepatotoxicity, which can lead to several clinical manifestation of liver disease. Some clinical studies have confirmed that the association of medicinal herbs containing *Scutellaria baicalensis Georgi* and *Bupleurum chinense DC* may induce liver fibrosis [239]. Some case reports in Japan and Taiwan have revealed that patients exhibited acute drug-induced liver injury after treatment with Xiao Chai Hu Tang, and *Scutellaria baicalensis Georgi* was believed to be one of the toxic ingredients in this herbal formula [240,241]. Animal experiments also indicated that long term administration of high dose wogonin (120 mg/kg) may lead to myocardial damage in rats [242]. Another study found that the ingredients containing *Scutellaria baicalensis Georgi* were detected at different stages of pregnancy in pregnant rats. This result demonstrated the potential risk of embryotoxicity may exist in *Scutellaria baicalensis Georgi* treatment, and further basic and clinical studies are urgently needed for this problem [243].

## 5. Conclusions

**Figure 2:**
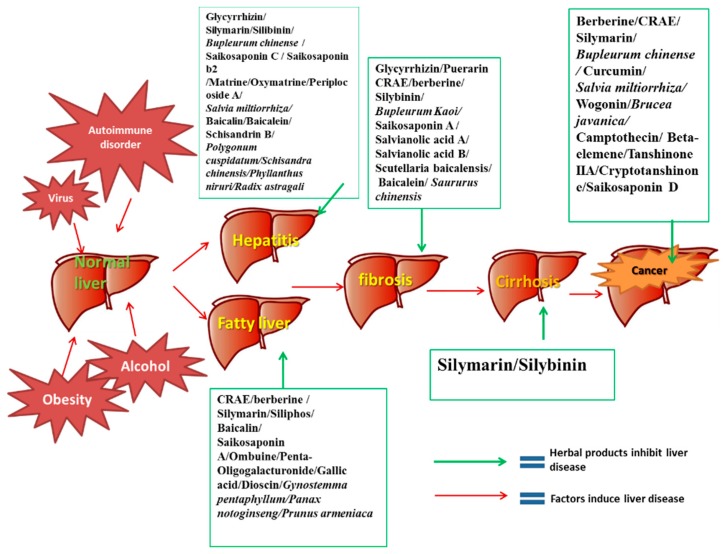
The roles of herbs and phytochemicals in the progression of chronic liver disease.

The current review provides a detailed and updated description of the most widely used herbal medicine and herbal formulas used in alleviating chronic liver disease. It has been clearly described that medicinal plants and phytochemicals can treat chronic liver disease by inhibiting oxidative damage, suppressing fibrogenesis, eliminating virus infection, and preventing or inhibiting tumor growth (Figure 2). For some medicinal plants, the active components still need to be further confirmed. More randomized, placebo-controlled clinical trials are urgently needed to confirm the clinical efficacy of this herbal medicine in chronic liver disease treatment. For the majority of medicinal herbs and phytochemicals, the safety investigation is just as important as the efficacy investigation. In future study, both basic research and clinical studies should be developed on the potential toxicity and side effects of these herbal medicines. More medicinal plants and phytochemicals with safe performance and significant efficacy are expected to be identified for use in chronic liver disease treatment in the future.
